# Regulation of constitutive and alternative mRNA splicing across the human transcriptome by PRPF8 is determined by 5′ splice site strength

**DOI:** 10.1186/s13059-015-0749-3

**Published:** 2015-09-21

**Authors:** Vihandha O. Wickramasinghe, Mar Gonzàlez-Porta, David Perera, Arthur R. Bartolozzi, Christopher R. Sibley, Martina Hallegger, Jernej Ule, John C. Marioni, Ashok R. Venkitaraman

**Affiliations:** The Medical Research Council Cancer Unit, University of Cambridge, Cambridge Biomedical Campus, Box 197, Cambridge, CB2 0XZ UK; European Molecular Biology Laboratory - European Bioinformatics Institute (EMBL-EBI), Hinxton, UK; Department of Molecular Neuroscience, UCL Institute of Neurology, Queen Square, London, WC1N 3BG UK

## Abstract

**Background:**

Sequential assembly of the human spliceosome on RNA transcripts regulates splicing across the human transcriptome. The core spliceosome component PRPF8 is essential for spliceosome assembly through its participation in ribonucleoprotein (RNP) complexes for splice-site recognition, branch-point formation and catalysis. PRPF8 deficiency is linked to human diseases like retinitis pigmentosa or myeloid neoplasia, but its genome-wide effects on constitutive and alternative splicing remain unclear.

**Results:**

Here, we show that alterations in RNA splicing patterns across the human transcriptome that occur in conditions of restricted cellular PRPF8 abundance are defined by the altered splicing of introns with weak 5′ splice sites. iCLIP of spliceosome components reveals that PRPF8 depletion decreases RNP complex formation at most splice sites in exon–intron junctions throughout the genome. However, impaired splicing affects only a subset of human transcripts, enriched for mitotic cell cycle factors, leading to mitotic arrest. Preferentially retained introns and differentially used exons in the affected genes contain weak 5′ splice sites, but are otherwise indistinguishable from adjacent spliced introns. Experimental enhancement of splice-site strength in mini-gene constructs overcomes the effects of PRPF8 depletion on the kinetics and fidelity of splicing during transcription.

**Conclusions:**

Competition for PRPF8 availability alters the transcription-coupled splicing of RNAs in which weak 5′ splice sites predominate, enabling diversification of human gene expression during biological processes like mitosis. Our findings exemplify the regulatory potential of changes in the core spliceosome machinery, which may be relevant to slow-onset human genetic diseases linked to PRPF8 deficiency.

**Electronic supplementary material:**

The online version of this article (doi:10.1186/s13059-015-0749-3) contains supplementary material, which is available to authorized users.

## Background

The splicing of nascent precursor messenger RNA (pre-mRNA) molecules to remove introns, and conjoin exons in different arrangements that potentially encode alternative protein isoforms, is fundamental for human gene expression (reviewed in [1, 2]). This process is carried out by the human spliceosome machinery, in which over 300 proteins sequentially assemble with uridine-rich small nuclear RNA molecules (U snRNAs) to form distinct small nuclear ribonucleoprotein complexes (snRNPs). The major spliceosome, comprising the U1, U2, U4, U5 and U6 snRNPs, executes >99 % of RNA splicing reactions in human cells [3]. This machinery recognizes pre-mRNA sequences at several motifs — the 5′ and 3′ splice sites, the branch point, and polypyrimidine tracts — positioned at exon–intron boundaries [4]. Stepwise sequential assembly of spliceosome components on these pre-mRNA motifs executes splicing reactions. One critical step involves recruitment of the pre-assembled U4/U6.U5 tri-snRNP to Complex A, which engages 5′ and 3′ splice sites, to form the pre-catalytic Complex B. Complex B then undergoes profound structural and conformational changes that lead to catalytic activation and conversion to Complex B^act^, which initiates catalysis and nucleates the formation of Complex C, which completes the splicing reaction [3, 4]. Remarkably, the major spliceosome accurately recognizes intended splice sites amidst a vast array of distinct exons and introns that, in metazoans, can range from hundreds to tens of thousands of base pairs in length [5]. Moreover, splice site recognition is flexible enough to allow the generation of a broad range of alternative splicing products [1]. *Trans*-acting splicing factors, pre-mRNA secondary structure and chromatin organization are now known to directly affect alternative splicing decisions [6]. Moreover, studies in yeast and other organisms suggest that depletion of core splicing factors can alter the splicing of subsets of genes [7–10]. However, it remains unclear what features of these genes dictate dynamic changes in their recognition and stepwise processing by spliceosome components to regulate splice site choice and splicing outcome.

The large ~280-kDa U5 snRNP protein PRPF8 is central to the dynamics of spliceosome assembly [11]. It not only makes direct contacts in the RNA substrate with the 5′ splice site, the branch point and the polypyrimidine tract in the 3′ splice site, but also engages the U5 and U6 snRNAs [12–20]. Mutations in human PRPF8 that disrupt these interactions, affecting spliceosome assembly and function, are found in autosomal dominant retinitis pigmentosa, a variable-onset hereditary disease that results in a progressively decreasing field of vision due to degeneration of rods and cones [21–26]. Loss-of-function mutants of human PRPF8 delay the assembly of spliceosome components on pre-mRNA, and impair the splicing of 9 % (5 of 57) of tested genes [24]. Moreover, PRPF8 depletion by RNA interference (RNAi) alters the pattern of alternative RNA splicing across 10–30 % of 96 known alternative splicing events [24]. Collectively, these observations raise the possibility that PRPF8 may differentially regulate the stoichiometry and dynamics of spliceosome assembly on different splice sites to modulate RNA splicing.

Here, we explore the genome-wide effects of human PRPF8 depletion on RNA splicing using spliceosome UV crosslinking and immunoprecipitation (iCLIP), which monitors the assembly of the spliceosome machinery on pre-mRNA molecules at single nucleotide resolution, combined with massively parallel sequencing of the transcriptome (RNA-seq) and computational analysis. Consistent with its role as a core spliceosome component that specifically cross-links with the 5′ splice site [13, 17], PRPF8 depletion decreases the interactions of the spliceosome machinery with 5′ splice sites in the majority of exon–intron junctions across the human genome. However, the decreased abundance of PRPF8 triggers changes in the constitutive and alternative splicing of only a subset of human transcripts with weak 5′ splice sites, including many essential for progression through the mitotic cell cycle, which leads to mitotic arrest. Notably, these effects are recapitulated via depletion of Complex B components that closely interact with PRPF8, whilst this is not the case for further Complex A and C components. Experimental enhancement of splice site strength using mini-genes encoding members of this subset restores comparable to wild type the kinetics and fidelity of splicing in conditions where PRPF8 abundance is limiting. Our findings suggest a model wherein competition for the core spliceosome component PRPF8 by RNA molecules with splice sites of different strength may control the stepwise assembly of spliceosome components to dictate the efficiency, kinetics and outcome of constitutive and alternative splicing in the human transcriptome, with implications for the control of biological processes like mitosis.

## Results

### Genome-wide decreases in spliceosome assembly at exon–intron junctions following PRPF8 depletion

To ascertain how PRPF8 abundance affects spliceosome assembly, we utilized iCLIP [27] to assess pre-mRNA interactions of snRNPs at single nucleotide resolution. Spliceosome iCLIP provides a global view of protein–RNA interactions of the spliceosome by employing immunoprecipitation of the small nuclear ribonucleoprotein polypeptides B and B1 (SNRPB, also known as SmB/B'), which are common to all spliceosomal snRNPs except U6 [28], under conditions that allow co-purification of other snRNP-associated RNA-binding proteins (see “Materials and methods” for full details). We performed spliceosome iCLIP on Cal51 cells depleted of PRPF8 using RNAi. PRPF8 expression was significantly decreased by >90 % in Cal51 cells exposed to a short interfering RNA (siRNA) targeting PRPF8, in comparison with a control siRNA, at the level of mRNA (Additional file 1a) as well as protein (Additional file 1b) expression.

**Fig. 1:**
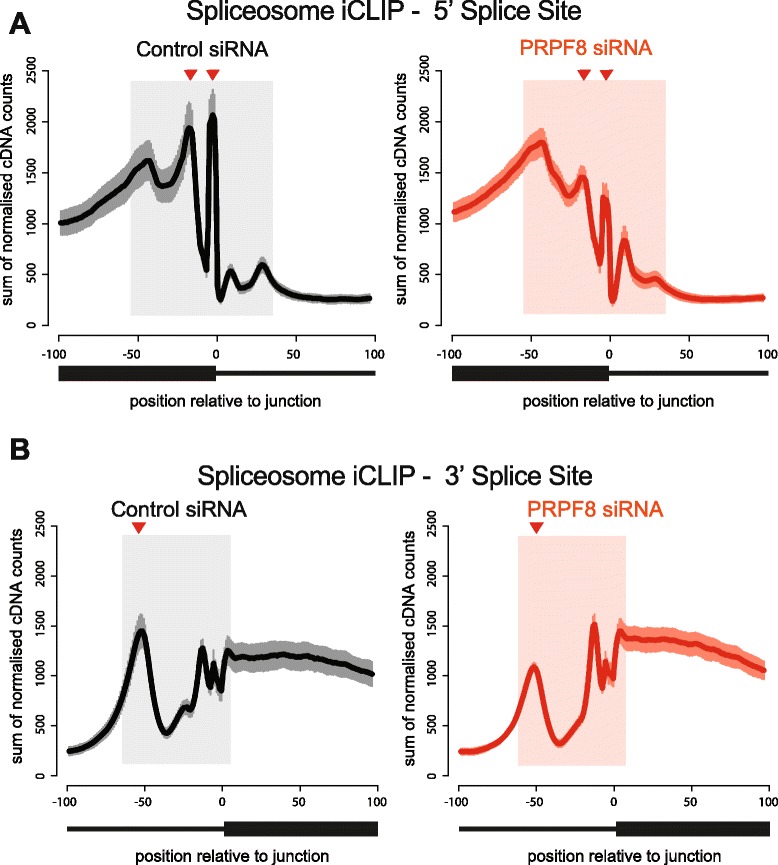
Genome-wide decreases in spliceosome assembly at exon–intron junctions following PRPF8 depletion. **a** Spliceosome iCLIP reveals spliceosomal interactions with pre-mRNA that change following PRPF8 depletion at the 5′ splice site. Spliceosome iCLIP cross-link sites summarized into genome-wide maps around splice junctions reveal distinct peaks associated with individual RNA-binding protein (RBP) occupancy. A large reduction in the amplitude of the cross-link peaks located ~20 nucleotides upstream of the 5′ splice site, and immediately adjacent to the exon–intron junction following PRPF8 depletion is indicated by *red arrowheads*. **b** Spliceosome iCLIP reveals spliceosomal interactions with pre-mRNA that change following PRPF8 depletion at the 3′ splice site. A reduction in the amplitude of the cross-link peak located ~50 nucleotides upstream of the 3′ splice site following PRPF8 depletion is indicated by *red arrowheads*

Spliceosome iCLIP cross-link sites summarized into genome-wide maps around splice junctions reveal distinct peaks associated with individual RNA-binding protein (RBP) occupancy. Changes at multiple positions are seen in response to PRPF8 depletion (Fig. 1a, b), each of which signifies interactions that occur relatively late during spliceosome assembly (J. Ule, unpublished observations; see “Materials and methods”). Most notable is the large reduction in the amplitude of the cross-link peak located ~20 nucleotides upstream of the 5′ splice site (Fig. 1a). This motif corresponds to the U5 snRNP binding site that tethers the 5′ exon following the first steps of splicing, and is consistent with the reported U5–PRPF8 interaction [29]. Importantly, although we observe a reduction in SmB/B’ protein levels following PRPF8 depletion, the amount of RNA going into each library from each immunoprecipitation reaction is equal, suggesting that the antibody is limiting rather than the starting material (Additional file 1c–e). Furthermore, the observed effects are unlikely to be the result of overall changes in gene expression following PRPF8 depletion as the distribution of a number of peaks associated with RBP occupancy are unchanged (for example, one peak located ~50 bp upstream of the 5′ splice site, and the majority of peaks at the 3′ splice site; Fig. 1b). Lastly, the detected differences persist even after excluding from the analysis genes that show significant differences in expression levels between the PRPF8-depleted and control samples (“Materials and methods”; Additional file 2a, b).

### Altered splicing after PRPF8 depletion affects only a subset of human transcripts

**Fig. 2:**
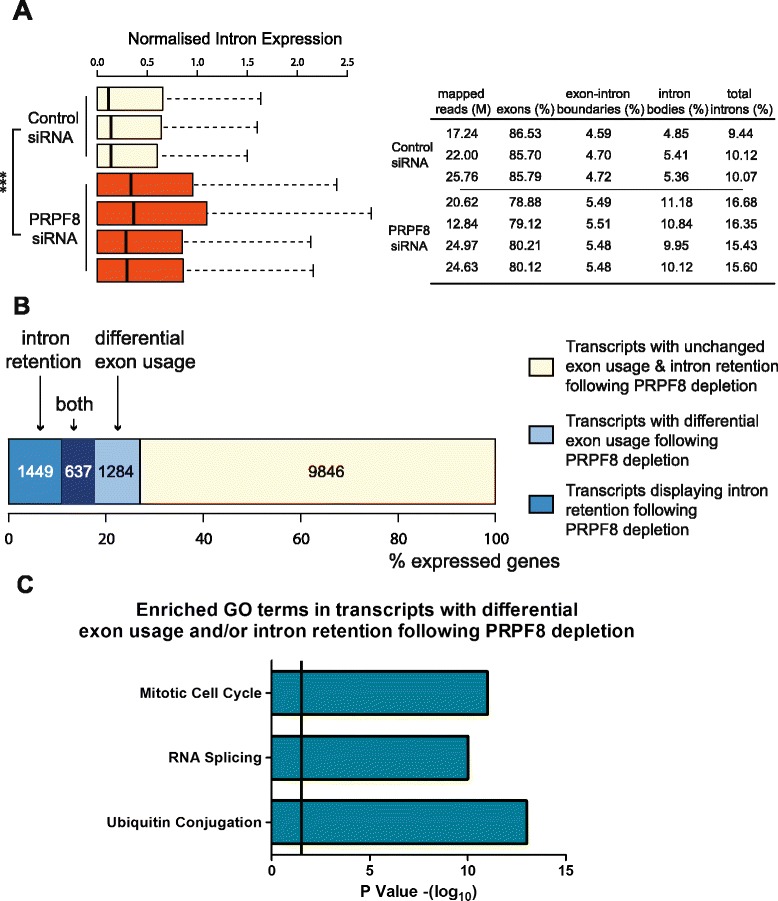
Altered splicing after PRPF8 depletion affects only a subset of human transcripts. **a** Intronic expression levels across the genome are increased in PRPF8-depleted cells. Normalized intron expression was calculated following analysis of the transcriptome of PRPF8-depleted Cal51 cells using RNA sequencing (*left*; “Materials and methods”). Statistically significant pairwise comparison is indicated (***p < 0.001). The number of mapped reads, the percentage of reads that map to exons, exon–intron boundaries, and intron bodies are shown in tabular form (*right*). **b** RNA sequencing experiments demonstrate that PRPF8 depletion results in altered splicing in only a subset of human transcripts. DEXSeq (“Materials and methods”) identifies a set of 2086 protein-coding genes that contain at least one retained intron [false discovery rate (FDR) < 0.01] and a set of 1921 protein-coding genes that display significant differences in exon usage (FDR < 0.01) following PRPF8 depletion; 637 genes display both retained introns and alternative exon usage (*p* < 2.2e-16). Transcripts with altered splicing patterns constitute only a subset of all expressed protein-coding genes (*n* = 3388 out of 13,216; expression threshold = 1 FPKM (Fragments per kilobase of exon per million reads mapped); “Materials and methods”). **c** Functional enrichment analysis using DAVID and WebGestalt (“Materials and methods”) shows that this subset is enriched for transcripts that participate in mitosis, ubiquitin conjugation, or RNA processing. *GO* Gene Ontology

Next, we analyzed the transcriptome of PRPF8-depleted Cal51 cells by massively parallel RNA sequencing (“Materials and methods”). Expression levels of intronic reads across the genome of PRPF8-depleted Cal51 cells are increased in comparison with controls (*p* value < 2.2e-16; Fig. 2a), consistent with the role of PRPF8 in regulating constitutive splicing. Interestingly, PRPF8 depletion significantly alters splicing in only a subset of human transcripts. We used DEXSeq (“Materials and methods”) to identify a set of 2086 protein-coding genes that contain at least one retained intron [false discovery rate (FDR) < 0.01] following PRPF8 depletion (Fig. 2b). We also executed DEXSeq on annotated exons (“Materials and methods”) to identify significant differences in exon usage in 1921 protein-coding genes following PRPF8 depletion (Fig. 2b; FDR < 0.01). Notably, there is a significant overlap between genes that harbor retained introns and exhibit alternative exon usage (*n* = 637; *p* value < 2.2e-16). Moreover, transcripts with altered splicing patterns constitute only a subset of all expressed protein-coding genes (*n* = 3370 out of 13,216; expression threshold = 1 FPKM (Fragments per kilobase of exon per million reads mapped); Fig. 2b; see “Materials and methods”).

We also asked if spliceosomal binding is specifically affected in the subset of introns that are preferentially retained following PRPF8 depletion. The observed changes in spliceosome iCLIP peaks around 5′ splice junctions in response to PRPF8 depletion are broadly similar in both the subset of retained introns (RI) and the larger subset of non-retained introns (NRI) (Additional file 2c). However, the spliceosome crosslinking pattern at the 3′ splice site changes more in the RI than the NRI subset (Additional file 2d). Indeed, we detect an increase in the crosslinking at the 3′ splice site intron–exon boundary in introns that are retained (Additional file 2d). One potential explanation may be that due to delayed tri-snRNP association, U2AF persists at the 3′ splice site, enhancing crosslinking that may be normally lost during spliceosome assembly, until the spliceosome recognizes the 3′ splice site again during exon ligation. Thus, the decreased spliceosome assembly we observe at 5′ splice sites in the majority of genomic intron–exon junctions after PRPF8 depletion may trigger more pronounced effects at 3′ splice sites in the RI subset of introns that are frequently retained when PRPF8 abundance is limiting. Alternatively, the increase in crosslinking at the 3′ splice site intron–exon boundary that we observe in introns that are retained may reflect a more direct role for PRPF8 in 3′ splice site recognition, consistent with cross-linking data [14–17].

### PRPF8 depletion impedes mitotic progression via altered splicing

Functional enrichment analysis on the subset of transcripts with altered splicing following PRPF8 depletion reveals that this subset is enriched for transcripts that participate in mitosis, ubiquitin conjugation, or RNA processing (Fig. 2c). To address the potential relevance of PRPF8-induced changes in RNA splicing on biological processes, we focused on genes encoding proteins with functions in mitosis, which were highly enriched in the subset that was sensitive to PRPF8 abundance (Fig. 2c). Indeed, we previously identified PRPF8 in an RNAi screen for factors that promote mitotic exit following drug-induced mitotic arrest [30]. Moreover, multiple spliceosome components, including PRPF8, were detected in an RNAi screen for factors affecting mitotic progression [31].

**Fig. 3:**
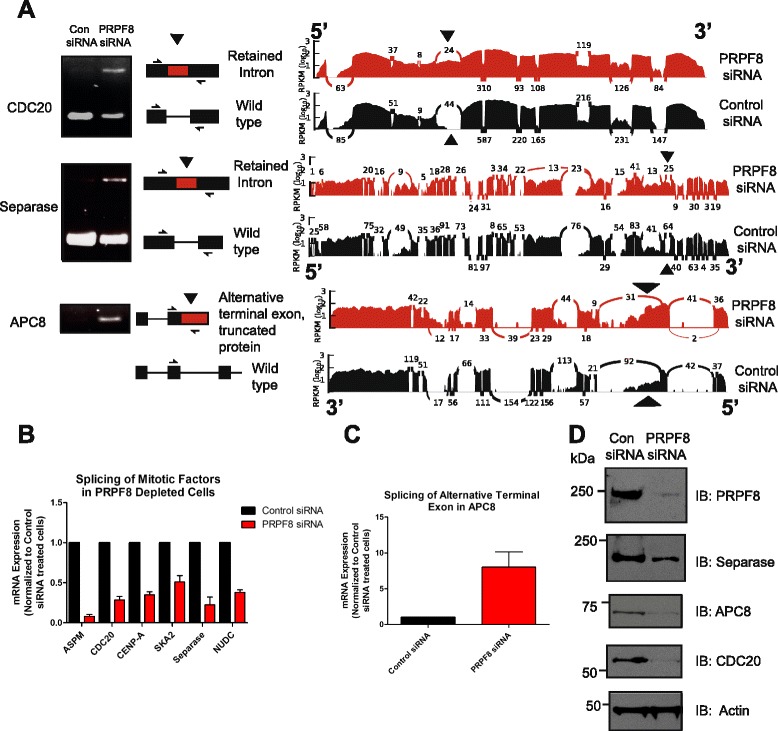
Altered splicing of transcripts encoding proteins required for mitotic progression after PRPF8 depletion. **a** RT-PCR analysis of RNA from PRPF8-depleted cells reveals a variety of splicing defects in transcripts that encode critical factors required for mitotic progression, including retained introns (*CDC20* and *Separase*), and alternative terminal exons (*APC8*). RT-PCR analysis of *CDC20*, *Separase*, and *APC8* using the primers indicated in the schematic are shown alongside the corresponding coverage plots (control siRNA in *black*, PRPF8 siRNA in *red*). The splicing defects are indicated by *arrowheads*. **b**, **c** Quantitative RT-PCR analysis of RNA from PRPF8-depleted cells, including retained introns (*CDC20*, *Separase*, and *NUDC*), skipped exons (*ASPM* and *SKA2*), and alternative terminal exons (*APC8*). Plots are relative to RNA levels in control siRNA-treated cells, assigned an arbitrary value of 1, and show the mean of triplicate readings from three independent experiments ± standard error of the mean. **d** Protein expression analysis of genes with altered splicing following PRPF8 depletion

Reverse transcription-polymerase chain reaction (RT-PCR) and quantitative RT-PCR (qRT-PCR) analysis of RNA isolated from PRPF8-depleted cells reveals a variety of splicing defects in transcripts that encode critical factors required for mitotic progression, including retained introns (e.g., *CDC20*, *Separase* and *NUDC*), skipped exons (e.g., *ASPM* and *SKA2*), and alternative terminal exons (e.g., *APC8*) (Fig. 3a–c; Additional file 3a). Importantly, these alterations do not normally occur during transit through mitosis in control cells (Additional file 3b, c). Furthermore, they manifest themselves at the protein level. For example, after PRPF8 depletion there is decreased expression of CDC20 and separase proteins (whose transcripts are enriched for retained introns and presumably destined for nonsense-mediated decay), as well as in the expression of full-length APC8 protein (whose transcripts are enriched for an alternative terminal exon) (Fig. 3d).

**Fig. 4:**
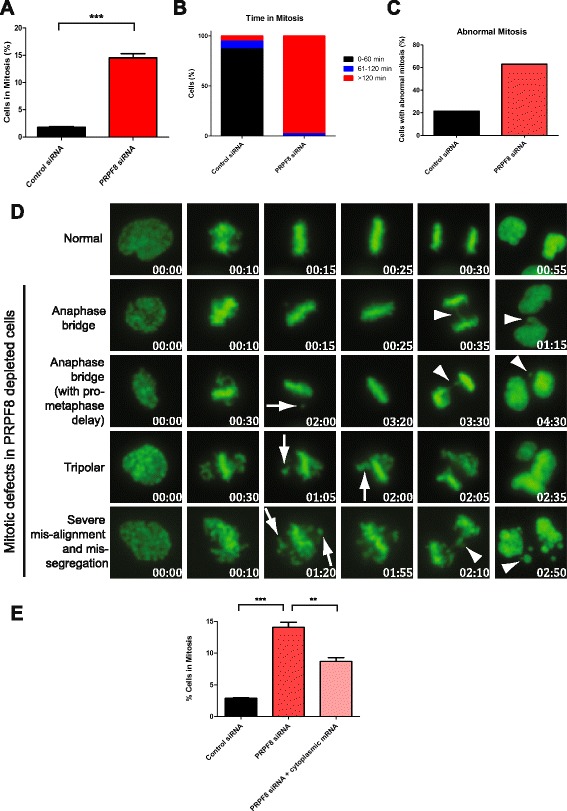
PRPF8 depletion impedes mitotic progression. **a** PRPF8 depletion in Cal51 cells results in a fivefold increase in the percentage of mitotic cells compared with control siRNA-treated cells. Plots show the mean from three independent experiments ± standard error of the mean. Statistically significant pairwise comparison is shown (***p < 0.001). **b** U2OS cells stably expressing green fluorescent protein (GFP)-tagged histone H2B, depleted of PRPF8, and monitored by phase-contrast time-lapse microscopy spend >120 min in mitosis, as opposed to control siRNA-treated cells, in which mitosis lasts, on average, <60 min. The bar graph depicts time spent in mitosis, measured from nuclear envelope breakdown to anaphase. **c**, **d** Live-cell imaging of U2OS GFP-H2B cells depleted of PRPF8 reveals multiple mitotic abnormalities, including unaligned chromosomes, anaphase bridges and multipolar divisions, indicated by arrows and arrowheads. Severe defects in both chromosome alignment and segregation are also observed in some cells. Representative images are shown in (**d**); defects are quantified in (**c**). **e** Introduction of properly spliced and processed mRNA into PRPF8-depleted cells suffices to partially rescue the observed mitotic defects. Control siRNA-treated and PRPF8-depleted cells were transfected with properly spliced and processed mRNA and analyzed for mitotic index. Plots represent the mean from three independent experiments ± standard error of the mean. Statistically significant pairwise comparisons are shown (**p < 0.01, ***p < 0.001)

Consistent with these effects, PRPF8 depletion in Cal51 cells results in a fivefold increase in the percentage of mitotic cells compared with controls (Fig. 4a). Moreover, U2OS osteosarcoma cells depleted of PRPF8 with two independent siRNAs and monitored by phase-contrast time-lapse microscopy spend >120 min in mitosis, as opposed to control cells, in which mitosis lasts, on average, <60 min (Fig. 4b; Additional file 4a). Live-cell imaging of U2OS cells stably expressing green fluorescent protein (GFP)-tagged histone H2B and depleted of PRPF8 reveals multiple mitotic abnormalities, including unaligned chromosomes, anaphase bridges and multipolar divisions (Fig. 4c, d). Severe defects in both chromosome alignment and segregation are also observed in some cells. Collectively, these defects are consistent with the observed mitotic delay. Furthermore, introduction of properly spliced and processed mRNA into PRPF8-depleted cells suffices to partially rescue the observed mitotic defects (Fig. 4e; Additional file 4b), suggesting that PRPF8 depletion affects mitotic progression via altered RNA splicing.

**Fig. 5:**
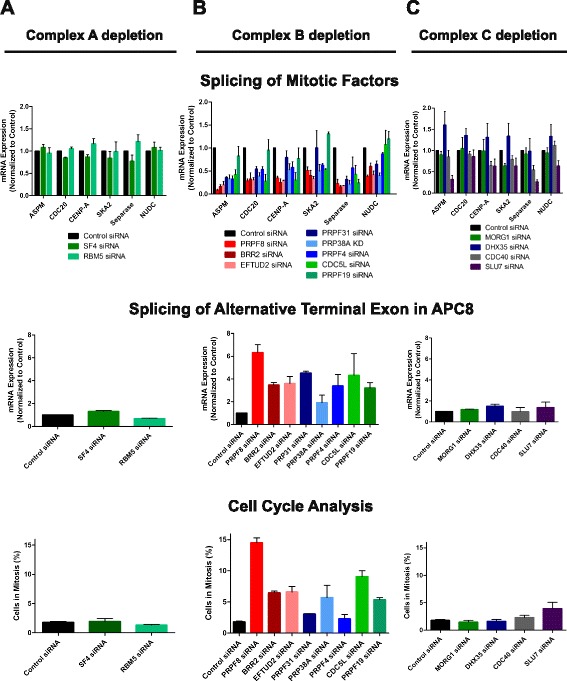
Depletion of Complex B components that interact directly with PRPF8 recapitulate defects in mitotic progression accompanied by the altered splicing of mitotic genes. **a** Depletion of Complex A components does not cause defects in either the splicing of mitotic genes (*top* and *middle panels*) or in mitotic progression (*bottom panel*). Independent depletion of several different components of the three major spliceosome subcomplexes (A, B, and C) was verified by qRT-PCR (Additional file 5). **b** Depletion of several Complex B components phenocopies PRPF8, in terms of both defects in splicing of mitotic genes (*top* and *middle panels*) and, to a lesser extent, increased mitotic index (*bottom panel*). Depletion of the spliceosome components BRR2 and EFTUD2, which interact directly with PRPF8 to form the U5 snRNP, most strongly phenocopy PRPF8 deficiency. **c** Depletion of Complex C components does not cause defects in either the splicing of mitotic genes (*top* and *middle panels*), or in mitotic progression (*bottom panel*). SLU7 partially phenocopies PRPF8 but does not display defects in mitotic progression. For all qRT-PCR experiments in this figure, plots are relative to RNA levels in control siRNA-treated cells, assigned an arbitrary value of 1, and show the mean of triplicate readings from at least three independent depletion experiments ± standard error of the mean (SEM). For cell cycle analysis experiments, plots represent the mean from at least three independent depletion experiments ± SEM

Supporting this notion, the depletion of certain additional spliceosome components closely associated with PRPF8 recapitulates defects in mitotic progression accompanied by the altered splicing of mitotic genes. Independent depletion of several different components of the three major spliceosome subcomplexes (A, B, and C) was verified by qRT-PCR (Additional file 5a). Depletion of several Complex B components phenocopies PRPF8, in terms of both defects in splicing of mitotic genes (Fig. 5a) and, to a lesser extent, mitosis (Fig. 5b; Additional file 5b, c). Notably, depletion of the spliceosome components BRR2 and EFTUD2, which interact directly with PRPF8 to form the U5 snRNP [32], most strongly phenocopy PRPF8 deficiency (Fig. 5b; Additional file 5b, c). In contrast, depletion of several Complex A or C components does not recapitulate the defects in splicing of mitotic genes and in mitotic progression observed following PRFP8 depletion (Fig. 5a, c; Additional file 5b, c).

### PRPF8 depletion induces preferential retention of introns, or inclusion of differentially used exons, with weak 5′ splice sites

**Fig. 6:**
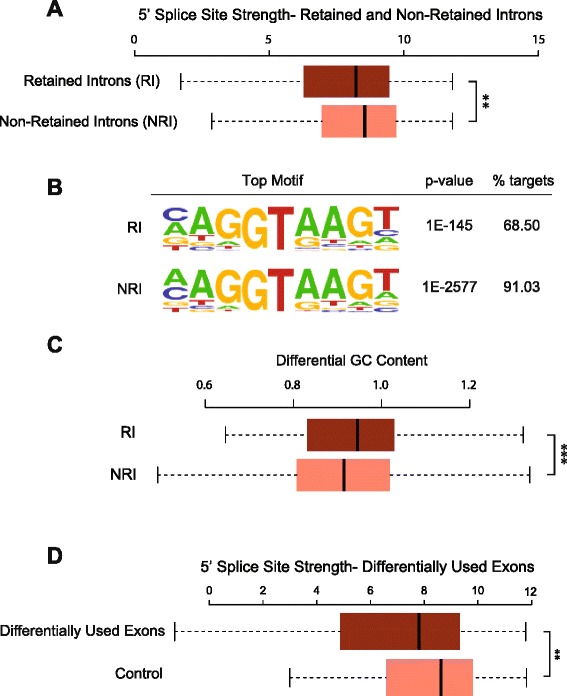
Introns that are retained and exons that are differentially used after PRPF8 depletion have weak 5′ splice sites. **a** Inefficiently spliced introns have weaker 5′ splice sites. A set of 200 retained introns (*RI*) were selected based on fold-change differences (see “Materials and methods”), and non-retained introns (*NRI*) within the same set of genes were used as a contrast. A 5′ splice site strength analysis was then carried out as described in “Materials and methods” for each subset. Statistically significant pairwise comparison is indicated (**p < 0.01). **b** Motif enrichment analysis on the same set of genes shows that although the most frequently identified motifs correspond to the consensus splice site sequences for both retained (RI) and non-retained introns (NRI), the percentage of targets with such motifs varies significantly between the two categories (68.50 % for RI versus 91.03 % for NRI; *p* < 2.2 × 10^−16^, Fisher’s exact test). **c** Analysis of GC composition reveals that retained introns have higher GC content than non-retained introns with values closer to those of adjacent exons. Differential GC content was calculated by dividing GC content of each intron to the average of its adjacent exons (see “Materials and methods”). Statistically significant pairwise comparison is indicated (***p < 0.001). **d** Differentially used exons have weaker 5′ splice sites. A 5′ splice site strength analysis was carried out as described in “Materials and methods” for each subset. Statistically significant pairwise comparison is indicated (***p < 0.001)

What shared characteristics might distinguish the subset of transcripts whose splicing is altered by PRPF8 depletion? To address this issue, we first evaluated possible differences in splice site strength between introns that are retained after PRPF8 depletion and those that are properly spliced, and we find that inefficiently spliced introns tend to have weaker 5′ splice sites (*p* value = 0.0220, Wilcoxon test; Fig. 6a; see “Materials and methods”). This finding is consistent with prior evidence suggesting that intron retention correlates with lower splice site strength [33]. Motif enrichment analysis on the same set of genes reveals consistent results (Fig. 6b); while the most frequently identified motifs correspond to the consensus splice site sequences for both RI and NRI subsets of genes, the percentage of targets with such motifs varies significantly between the two categories (68.50 % for RI versus 91.03 % for NRI; *p* value < 2.2 × 10^−16^, Fisher’s exact test). Notably, such differences are not observed at the 3′ splice site (Additional file 6a, b), which is consistent with data suggesting that yeast PRPF8 cross-links most strongly to the 5′ splice site [13, 17, 19]. In addition, analysis of GC composition reveals that retained introns have slightly higher GC content than non-retained introns (*p* value = 0.0055, Wilcoxon test; Fig. 6c), with values closer to those of adjacent exons (*p* value < 2.2 × 10^−16^, Wilcoxon test; Fig. 6). This result agrees with previous findings suggesting that introns with GC content similar to that of adjacent exons are more likely to be retained [34]. Next, we determined whether differences in splice site strength in differentially used exons could also explain the increase in alternative splicing events that we observe following PRPF8 depletion. Consistent with the results detected for intron retention events, differentially used exons harbor weaker 5′ splice sites (Fig. 6d; Additional file 6c).

### Enhancement of splice-site strength renders mini-genes resistant to PRPF8 depletion

**Fig. 7:**
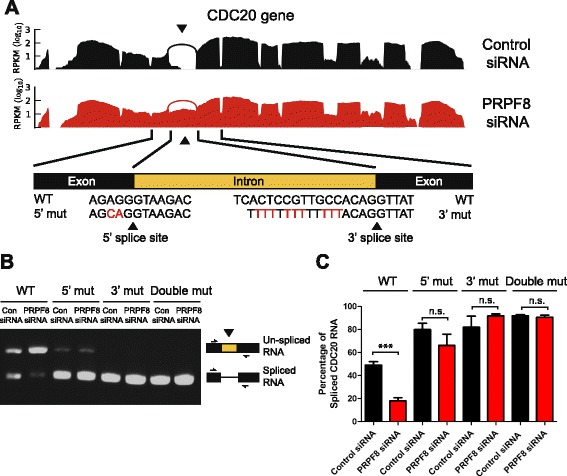
Enhancement of splice-site strength renders the *CDC20* mini-gene resistant to PRPF8 depletion. **a** A mini-gene containing a full-length intron flanked by two exons within the *CDC20* gene. This particular intron is retained following PRPF8 depletion as determined by RNA-sequencing experiments and the corresponding coverage plot is shown. Both the 5′ splice site and the 3′ splice site encompassing the exon–intron and intron–exon boundaries are weaker than the corresponding consensus sequences and the sequence of 5′ and 3′ splice mutant constructs are indicated with changes marked in *red. RPKM* Reads per kilobase of exon per million reads mapped, *WT* wild type. **b, c** Enhancement of splice site strength renders the *CDC20* mini-gene resistant to PRPF8 depletion. PRPF8 depletion strongly suppresses removal of this intron in the *CDC20* mini-gene (*left panel*). Alteration by site-directed mutagenesis of the weak 5′ splice site into a strong consensus sequence leads to efficient removal of this intron in PRPF8-depleted cells, in a manner indistinguishable from control siRNA-treated cells (*middle panel*). Strengthening of the polypyrimidine tract also allows efficient removal of this intron from the *CDC20* mini-gene in PRPF8-deficient cells (*middle panel*). Plots in (**c**) represent quantification of band intensity of spliced and unspliced product using ImageJ (NIH) and represent the mean percentage of spliced mRNA from three independent experiments ± standard error of the mean. Statistically significant pairwise comparison is indicated (***p < 0.001); *n.s*. not significant

To confirm that splice-site strength determines the outcome of RNA splicing under conditions where PRPF8 availability is limiting, we generated a mini-gene containing a full-length intron flanked by two exons within the *CDC20* gene. This particular intron is retained following PRPF8 depletion as determined by RNA-sequencing experiments and validated by RT-PCR (Figs. 2a and 7a). Indeed, both the 5′ splice site and the 3′ splice site encompassing the exon–intron and intron–exon boundaries are weaker than the corresponding consensus sequences (Fig. 7a). As predicted, PRPF8 depletion strongly suppresses removal of this intron in the *CDC20* mini-gene (Fig. 7b, c). Importantly, however, alteration by site-directed mutagenesis of the weak 5′ splice site into a strong consensus sequence leads to efficient removal of this intron in PRPF8-depleted cells, in a manner indistinguishable from control siRNA-treated cells (Fig. 7b, c). Furthermore, strengthening of the polypyrimidine tract also allows efficient removal of this intron from the *CDC20* mini-gene in PRPF8-depleted cells (Fig. 7b, c; Additional file 7a–c). Indeed, a number of studies have suggested that strong 3′ splice sites can compensate for weak 5′ splice sites [35, 36], although this may also reflect a role for PRPF8 at the 3′ splice site, consistent with cross-linking data [14–17]. Thus, the enhancement of splice site strength renders mini-genes resistant to mis-splicing provoked by PRPF8 depletion, suggesting that competition for PRPF8 availability may dictate genome-wide patterns of RNA splicing.

### PRPF8 depletion alters the dynamics of RNA splicing during transcription

Collectively, our computational and experimental findings suggest that PRPF8 has a rate-limiting function in the splicing of introns with weak splice sites during RNA transcription, leading to alternative RNA splicing patterns in conditions where PRPF8 abundance is restricted. To explore the mechanism behind this function, we analyzed changes in the dynamics of RNA splicing after PRPF8 depletion using two methods. The first method evaluates splicing dynamics by comparatively enumerating the number of intronic reads in different genes detected by massively parallel RNA sequencing. The second utilizes a previously described approach that measures the time between synthesis of a new exon and the appearance of the splicing product of that exon following removal of a reversible transcription inhibitor [37].

**Fig. 8:**
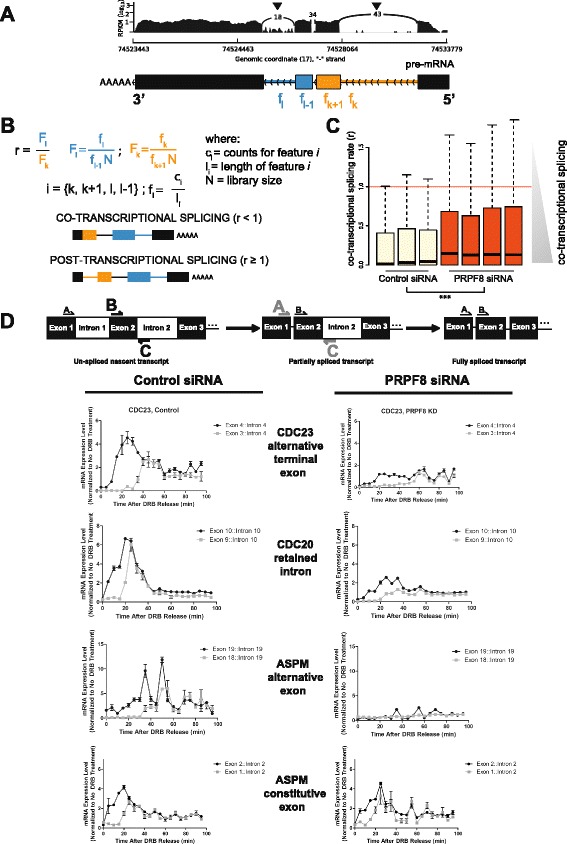
PRPF8 depletion alters the dynamics of RNA splicing during transcription. **a**, **b** Intronic reads contain potential information on the dynamics of splicing. Introns located towards the 5′ end of transcripts have a higher probability of being spliced out under a scenario of co-transcriptional splicing, but this is not the case if splicing occurs post-transcriptionally. A co-transcriptional splicing ratio was calculated by comparing the intronic coverage of the first versus last introns in each transcript, as indicated in (**b**), with the values used to calculate the ratio shown schematically in (**a**). Such coverage was normalized to take into account intron length and the expression levels of adjacent exons. A representative example is shown in (**a**), with first and last intronic reads indicated by *arrowheads. RPKM* Reads per kilobase of exon per million reads mapped. **c** Distribution of the co-transcriptional splicing ratio in control siRNA-treated and PRPF8-depleted samples. Co-transcriptional splicing dominates in control siRNA-treated samples as indicated by the low ratio values. The co-transcriptional splicing ratio significantly increases following PRPF8 depletion, suggesting disruptions in the co-transcriptional splicing efficiency (*p* < 5.34 × 10^−9^). Only genes with one transcript annotated in Ensembl 66 were considered for this analysis (*n* = 2366; “Materials and methods”). **d** PRPF8 depletion alters the dynamics of RNA splicing during transcription. The kinetics of transcription and splicing recovery of *CDC20*, *APC8*, and *ASPM* genes following release from drug-induced transcriptional arrest were measured in control siRNA-treated and PRPF8-depleted cells using a previously published protocol [37]. Primer pairs used are indicated and were chosen to measure the dynamics of RNA splicing of introns that are either retained or exons that are differentially used as determined by RNA sequencing experiments. As a control, the kinetics of splicing of an exon that was not differentially used in the ASPM transcript was also measured. PRPF8 depletion inhibits the kinetics of splicing as the delay between detection of the unspliced nascent transcript (indicated in *black*) and the partially spliced transcript (indicated in *light gray*) is ~20 minutes for *CDC23* and ~15 minutes for *ASPM* in control siRNA-treated cells, but in excess of 40 minutes in PRPF8-depleted cells. In contrast, the kinetics of splicing and removal of intron 1 in the ASPM transcript is similar in both conditions. mRNA expression levels were normalized to non-DRB-treated cells for each condition (control siRNA-treated and PRPF8-depleted cells), which were harvested alongside the cells just released from DRB (0 minute time-point). *DRB* 5,6-dichlorobenzimidazole 1-β-D-ribofuranoside

Using our RNA sequencing data for poly(A)-selected transcripts from PRPF8-depleted and control siRNA-treated cells enabled analysis of the dynamics of the splicing reaction from intronic reads. Numerous studies in multiple organisms indicate that splicing is coupled to transcription [37–43]. Accordingly, we comparatively enumerated sequence reads mapped to the first and last introns for each transcript in PRPF8-depleted versus control siRNA-treated cells, in order to measure the extent to which co-transcriptional splicing had been perturbed following the depletion of a core spliceosomal component (Fig. 8a). Specifically, under a scenario of transcription-coupled splicing, introns located towards the 5′ end of transcripts are the first to be removed, and hence are less likely to be present in the transcript by the time poly(A) tailing occurs. Conversely, introns located towards the 3′ end are more likely to be detected, since their removal may not be completed when polyadenylation takes place. Consistent with our hypothesis, we observe that introns located towards the 3′ end of transcripts are expressed at higher levels than those situated towards the 5′ end (Fig. 8a, b; Additional file 8a). Importantly, weak splice sites are not enriched in the first introns in the set of 2380 genes considered for the analysis of the co-transcriptional splicing ratio, as observed when plotting the 5′ splice site strength for the first versus last introns (Additional file 8b). This provides additional evidence to support the theory that splicing occurs predominantly in concert with transcription [38]. Moreover, the calculated ratio significantly increases following PRPF8 depletion, suggesting that changes in the abundance of this core spliceosomal component can reduce co-transcriptional splicing efficiency (Fig. 8c; *p* value = 5.34 × 10^−9^).

To experimentally validate these observations, we measured the kinetics of transcription and splicing recovery following release from drug-induced transcriptional arrest [37]. Briefly, control siRNA-treated cells and PRPF8-depleted cells were treated with DRB (5,6-dichlorobenzimidazole 1-β-D-ribofuranoside), a reversible inhibitor of transcriptional elongation [44]. Total RNA was then harvested at defined time-points following DRB removal and the time between synthesis of a new exon and the appearance of the splicing product of that exon was measured [37]. We analyzed the kinetics of splicing in a set of four mitotic genes (*ASPM*, *CDC20*, *CDC23*, and *Separase*), in which we had previously identified intron retention and differential exon usage following PRPF8 depletion (Fig. 2). Broadly consistent with published work [37], we find that all of the introns tested were spliced and removed within 5–20 minutes of transcription of the downstream exon in control siRNA-treated cells (Fig. 8d; Additional file 7c). Interestingly, PRPF8 depletion affects the kinetics of splicing and intron removal (Fig. 8d; Additional file 8c). Thus, the delay between detection of the unspliced nascent transcript and the partially spliced transcript is ~20 minutes for *CDC23* and ~15 minutes for *ASPM* in control siRNA-treated cells, but in excess of 40 minutes in PRPF8-depleted cells (Fig. 8d). In contrast, the kinetics of splicing and removal of intron 1 in the ASPM transcript (whose intron 19 is affected as shown in Fig. 8) is similar in control siRNA-treated and PRPF8-depleted cells (Fig. 8d). The delay between detection of the unspliced nascent transcript and the partially spliced transcript is ~15 minutes for ASPM in both control siRNA-treated and PRPF8-depleted cells, and importantly the absolute recovery of transcription and splicing is also similar (Fig. 8d). Furthermore, the delay between detection of the unspliced nascent transcript and the partially spliced transcript for *UBE2R2*, a gene whose splicing was unaffected by PRPF8 depletion as determined in our RNA-sequencing data, is similar in both PRPF8-depleted and control siRNA-treated cells (Additional file 9a). For all genes tested, there is recovery of transcription following release from DRB in both PRPF8-depleted and control siRNA-treated cells, but the absolute recovery is markedly lower following PRPF8 depletion. Therefore, to rule out the possibility that the transcription machinery itself is affected by PRPF8 depletion, we measured transcription of the first exon–intron junction of the four target genes following release from DRB in PRPF8-depleted and control siRNA-treated cells, and found that the relative rate of transcription elongation is similar in both conditions (Additional file 9b). Thus, taken together, our results provide multiple lines of evidence that PRPF8 has a rate-limiting function in the splicing of introns with weak 5′ splice sites during RNA transcription, leading to alternative RNA splicing patterns in a subset of human genes, including those encoding several mitotic cell cycle proteins, when PRPF8 abundance is restricted.

## Discussion and Conclusions

Our results provide fresh insight into the mechanisms that enable the human spliceosome to recognize and sequentially assemble on intended splice sites within a vast array of transcribed pre-mRNA sequences. We show that restricting the abundance of PRPF8 — a key spliceosome component that bridges ribonucleoprotein complexes mediating splice-site recognition and catalysis — preferentially alters the splicing of a subset of human genes that predominantly contain weak 5′ splice sites. Although studies in yeast and other organisms have previously suggested that splicing of subsets of genes can be affected by depletion of various core splicing factors [7–10], we identify weak 5′ splice sites as one defining characteristic of the subset of introns affected by restricted PRPF8 abundance. By using spliceosome iCLIP, we demonstrate that the specificity of PRPF8 depletion on splicing events strikingly correlates with both weaker 5′ splice sites as well as decreased interaction of the spliceosome with 5′ splice sites. Interestingly, this subset is highly enriched for genes that encode factors essential for RNA processing, ubiquitin conjugation, or mitotic cell cycle progression, raising questions concerning the functional significance of their altered splicing. Indeed, we find that PRPF8 depletion provokes mitotic arrest in dividing cells, accompanied by intron retention and alternative exon usage in several mitotic genes, along with reduced abundance of their encoded proteins. Moreover, we demonstrate that these alterations are ameliorated by the experimental enhancement of splice site strength in mini-genes encoding affected introns in mitotic genes. Lastly, we show that PRPF8 depletion alters the dynamics of splicing during transcription. Our findings have several interesting implications.

We provide additional evidence supporting the idea that splicing is largely coupled to transcription. This comes from computational analysis of intronic reads, as well as experimental analyses. Our computational analysis is consistent with previous genome-wide observations that were based on the analysis of junction reads [43], and indicate that it is possible to gain biological knowledge from the intronic data generated in RNA-sequencing experiments, which are typically not considered for analysis. Notably, the transcription-coupled splicing ratio is significantly increased following PRPF8 depletion and a number of transcripts display a ratio >1. In these transcripts, intronic reads for the first intron are greater than those for the last intron, suggesting that there is an increased likelihood that these particular transcripts are spliced post-transcriptionally. This may result in increased competition for upstream and downstream RNA sequence elements by the spliceosome, such that strong splice sites would be favored over weak ones, especially in conditions where spliceosome assembly and activation is suboptimal, for example, following PRPF8 depletion. Thus, a potential change from co-transcriptional to post-transcriptional splicing may have an effect on the outcome of the splicing reaction. Taken together with the kinetics of splicing and intron removal experiments, our findings suggest that reductions in PRPF8 levels may in part uncouple transcription from splicing, allowing more time for introns with weak splice sites to be removed.

**Fig. 9:**
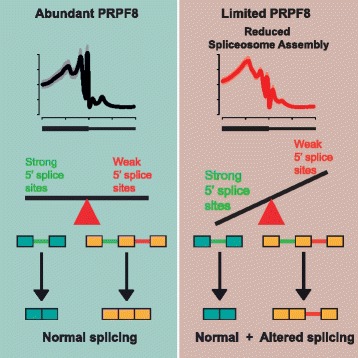
Model describing role of 5′ splice site strength in determining constitutive and alternative splicing regulated by PRPF8. In conditions of abundant PRPF8, there is competition between strong and weak 5′ splice sites to determine splice site choice and spliceosome assembly is proficient. When PRPF8 is limited, this causes a reduction in spliceosome assembly, such that strong 5′ splice sites are favored over weak 5′ splice sites, resulting in altered splicing patterns in a subset of transcripts

Consistent with the observations derived from the spliceosome iCLIP data, RNA-sequencing experiments show an overall increase in intronic expression levels. However, defective splicing is detected in only a subset of introns, which present typical characteristics of retained introns, including weaker 5′ splice sites [45], and lower differential GC content [34]. Indeed, PRPF8 is one of the few spliceosomal components that can directly contact the 5′ splice site, as well as the branch point and part of the polypyrimidine tract in the 3′ splice site region [13–20]. In yeast, its interaction with the 5′ splice site has been reported as being stronger [19], consistent with both our spliceosome iCLIP results and our splice site strength and motif analysis. Altogether, these findings suggest that the splicing alterations we observe are likely to arise from changes in the equilibrium reached between weak and strong splice sites in their competition for binding to the spliceosome, such that stronger 5′ splice sites are favored when PRPF8 abundance is limiting. Thus, although spliceosome assembly at 5′ splice sites is affected genome-wide, a decrease in spliceosomal binding at strong 5′ splice sites would not compromise splicing of the associated introns. We have validated this hypothesis with mini-gene experiments, in which we show that experimental enhancement of splice site strength can restore the kinetic competition equilibrium following PRPF8 depletion. On the other hand, although one of the major phenotypes that we observe in PRPF8-depleted cells is an increase in intron retention, we also detect an increase in alternative exon usage, raising the possibility that competition for binding to PRPF8 may regulate alternative splicing (Fig. 9). Indeed, we also detect weaker 5′ splice sites for differentially used exons in PRPF8-depleted cells. This is consistent with evidence suggesting that changes in the concentration of core components of the spliceosome such as SmB/B’ can also regulate this process [9, 24, 46].

The reduction in cross-linking at 5′ splice sites after PRPF8 depletion that we observe from our spliceosome iCLIP data is expected to reflect a decrease in snRNP association with the pre-mRNA. An alternative hypothesis is that PRPF8 depletion leads to a different conformation of the bound snRNPs that does not allow efficient cross-linking. The iCLIP protocol is unable to directly evaluate this in its current format since no structural information about the RBPs being precipitated is provided by this technique. Arguing against this alternative hypothesis, PRPF8 is not required for a minimal U1 snRNP to be formed [47], whilst a recent study in yeast found that deleting the sites of direct interaction between the U1 snRNA with Prp8 had no affect on both U1 snRNA levels or the protein components of the U1 snRNP [19]. Collectively this suggests that PRPF8 is not required for U1 snRNP formation and cross-linking, and that the reduced signal in our experiments most likely results from reduced association of the U1 snRNP with the pre-mRNA targets, rather than through a conformational change resulting from PRFP8 depletion. However, disrupting the direct interaction between U1 snRNP and Prp8 in yeast did reduce recruitment of the U4/U6.U5 tri-snRNP and the U2 snRNP during the splicing cycle, possibly explaining reduced cross-linking at the 3′ splice site [19]. The mechanisms behind this still remain unclear.

Our findings are consistent with previous reports that reductions in the activity or concentration of core spliceosome components can lead to alterations in the kinetics of spliceosomal rearrangements and differences in splice site choice [2, 48–53]. It is important to note that siRNA-mediated depletion never leads to a complete depletion of the protein of interest, so in our experiments there will still be residual PRPF8 protein present in the cells, albeit at a low level (>90 % reduction in mRNA and protein levels; Additional file 1). This is supported by our iCLIP data, which show that PRPF8 depletion results in a large reduction, but not complete abolition, of the cross-link peak expected to correspond to the U5 snRNP binding site located ~10–20 nucleotides upstream of the 5′ splice site (Fig. 1c). Thus, spliceosome assembly/activation is likely to be severely compromised, but not completely abrogated in PRPF8-depleted cells. Importantly, our results suggest that components of Complex B, and more specifically BRR2 and EFTUD2, which interact directly with PRPF8 to form the U5 snRNP complex [32], phenocopy PRPF8 most strongly. Indeed, spliceosome activation and disassembly are regulated by both EFTUD2 and BRR2 [54]. Depletion of several Complex A or C components does not recapitulate the defects in splicing of mitotic genes and in mitotic progression observed following PRFP8 depletion, suggesting that it is the U5 snRNP which is critical for regulating spliceosome dynamics and the removal of introns that are difficult to splice.

Expression levels of core spliceosome factors can vary during development, across tissue types and in disease [10, 55]. Importantly, mutations in both PRPF8 and BRR2 are found in autosomal dominant retinitis pigmentosa [21–26]. It is unclear why mutations in broadly expressed genes encoding core spliceosomal components result in a highly tissue-specific pathology with no apparent effects on other cell types. However, several studies have demonstrated that the expression levels of U5-snRNP proteins as well as levels of splicing are higher in the retina [24, 56, 57]. Furthermore, it has been suggested that mutations in U5 snRNP components affect spliceosome assembly and function and impair the splicing of 9 % (5 of 57) tested genes [24], although the specific target transcripts are unknown. It remains to be seen whether mutations in core spliceosomal components like PRPF8, as seen in autosomal dominant retinitis pigmentosa, also change the kinetic competition equilibrium between weak and strong splice sites, just as we observe when PRPF8 is depleted. Such a change may only result in subtle changes in expression of a subset of genes, but the cumulative effect of a small change may manifest over a number of years, consistent with the fact that retinitis pigmentosa is a gradual-onset disease [58]. Indeed, two recent studies reported that a number of myeloid neoplasms contain somatic PRPF8 mutations or hemizygous deletions with associated splicing defects [59] and a role for PRPF8 in the splicing of genes required for sister chromatid cohesion [60]. Thus, the model for spliceosome regulation suggested by our findings (Fig. 9) has implications for disease pathogenesis that warrant future investigation.

## Materials and methods

### Cell culture

Cal51 breast adenocarcinoma and U2OS osteosarcoma cells were cultured in Dulbecco’s modified Eagle medium (DMEM; Invitrogen) with 10 % fetal calf serum (Invitrogen) and 1× penicillin-streptomycin (Invitrogen).

### siRNA-mediated depletion, antibodies and western blotting

For siRNA-mediated depletion, Cal51 cells were reverse transfected with 25 nM siRNA to the indicated genes using DharmaFECT1 (Dharmafect) transfection reagent. Transfected cells were harvested 54 hours later for RNA extraction, western blotting and fluorescence-activated cell sorting (FACS). Efficiency of depletion was either monitored by western blotting with the indicated antibodies or by qRT-PCR against the indicated genes. Antibodies used were anti-PRPF8 (Abcam), anti-Separase (Abcam), anti-CDC20 (Santa Cruz), anti-APC8 (Novus Biologicals), anti-Actin (Abcam).

### RNA extraction, RT-PCR and qRT-PCR

RNA was isolated from siRNA-treated cells with an RNeasy kit (Qiagen) according to the manufacturer’s instructions. Isolated RNA was quantified with a NanoDrop 1000 (Thermo Scientific) and quality was determined by measuring the A_260_/A_280_ ratio, which was always between 1.8 and 2.1. All RNA was stored at −80°C. RNA (1 μg) was used for cDNA synthesis using the QuantiTect Reverse Transcription Kit (Qiagen) according to the manufacturer’s instructions. Synthesized cDNA was diluted following reverse transcriptase inactivation and stored at −20°C. Primers for qPCR were designed to bridge exon–intron junctions in cases of depletion validation. For mitosis targets, they were designed to bridge mis-spliced portions of transcripts as determined by RNA-sequencing experiments in PRPF8-depleted cells. For RT-PCR experiments, PCR was conducted on a MJ Research thermal cycler using Accuprime Pfx DNA polymerase (Invitrogen), forward and reverse primer, and cDNA. qPCR was conducted on either Rotorgene RG-3000 (Corbett Research) or Light Cycler 480 (Roche) machines using 2× SYBR-Green Master Mix (Roche or Invitrogen), forward and reverse primer, and cDNA. The cycling acquisition program was as follows: 50 °C 2 minutes, 95 °C 2 minutes, 50 cycles of 95 °C for 15 seconds and 60 °C for 30 seconds. The C_t_ values were calculated, referenced to standard curves for each primer set. All samples were then normalized to control siRNA-treated samples. Primer sequences are available on request.

### Live cell imaging

For live-cell imaging, U2OS cells were reverse transfected with 25 nM siRNA oligos onto multi-well 1.0-mm-thick borosilicate chamber slides (Lab-Tek) using DharmaFECT-1 (Thermo Scientific). Phase-contrast, multi-point time-lapse microscopy was performed on a Zeiss Axiovert 200M microscope, acquiring images every 3 minutes under a 20× objective. To visualize chromatin, GFP-tagged histone H2B was subcloned from pH2B-GFP (Addgene plasmid 11680) into pcDNA3.1/puro (a kind gift from Chris Sullivan, University of Texas). The H2B-GFP transgene was then stably integrated into U2OS cells, which were selected with 1 μg/ml puromycin (Invitrogen). Fluorescence live-cell imaging was performed on a Leica DMI6000 microscope using a HCX PL APO objective (40× magnification, N.A. 0.85), and images acquired every 5 minutes on an Evolve 512 EMCCD camera (Photometrics) using LAS AF software (Leica), then processed and analyzed with ImageJ (NIH).

### Flow cytometry

For determination of mitotic index, cells were fixed in ethanol, blocked in phosphate-buffered saline (PBS) with 0.1 % Triton X-100 (PBST) and 1 % bovine serum albumin for 30 minutes, then incubated with mouse anti-phospho-MPM2 antibody 1:500 (Millipore) for 2 hours at room temperature. Following washes with blocking solution, cells were incubated with Alexa Fluor 488 secondary antibody (Life Technologies) for 1 hour, washed with PBST and treated with 40 μg/ml propidium iodide (Sigma) and 200 μg/ml RNaseA (Sigma) before analyzing on a Becton Dickinson LSR II flow cytometer. Some cells were treated with 200 ng/ml of nocodazole (Sigma) for 4 or 15 hours to induce mitotic arrest prior to harvest.

### Rescue experiments with fully spliced poly(A)+ mRNA

Poly(A)+ mRNA was purified from the cytoplasm of cultured cells using OligoTex kit (Qiagen) according to the manufacturer’s protocol. mRNA (2 μg) was then transfected into cells which had been previously transfected 19 hours earlier with control siRNA or PRPF8 siRNA using TransMessenger transfection reagent (Qiagen) according to the manufacturer’s instructions. Cells were harvested 31 hours post-transfection (50 hours in total) and analyzed for mitotic index by flow cytometry as above. Data represent the mean from three independent experiments ± standard error of the mean (SEM).

### Mini-gene experiments

We generated a mini-gene containing a full-length intron flanked by two exons within the *CDC20* gene using PCR to amplify the respective region from genomic DNA using Accuprime Pfx (Invitrogen) to avoid the introduction of point mutations. This particular intron is retained following PRPF8 depletion as determined by RNA-sequencing experiments and validated by RT-PCR (Fig. 7a). This sequence was cloned into pcDNA3 (Invitrogen) using BamHI and ApaI restriction enzymes. To make the 5′ splice site mutant, QuikChange II XL Site Mutagenesis Kit (Stratagene) was used to generate the following mutation (mutated nucleotides are underlined): 5′- CCACAAAATGCGCCAGCAGGTAAGACCCGAAGTTC. To strengthen the polypyrimidine tract, we generated a shortened PCR product using either the 5′ primer: GCGTCTAGACTTTTTTTTTTTTTACAGGTTATCAGAACAGACTG, GCGTCTAGACTCACTCCGTTTCCACAGGTTATCAGAACAGACTG, GCGTCTAGACTCACTCCTTTTCCACAGGTTATCAGAACAGACTG or GCGTCTAGACTCACTCCGTTTTTACAGGTTATCAGAACAGACTG. This product was just downstream of an internal XbaI site, and was subcloned into either the full-length vector (to generate a 3′ splice mutant) or the 5′ splice site mutant vector (to generate a double mutant) using XbaI and ApaI restriction enzymes. All constructs were verified by sequencing. These constructs were transfected into cells that had been transfected 24 hours earlier with either control siRNA or PRPF8 siRNA using JetPrime transfection reagent (Polyplus). Total RNA was harvested from cells 30 hours post-transfection with plasmids (54 hours in total) and 1 μg of RNA was used for cDNA synthesis using the QuantiTect Reverse Transcription Kit (Qiagen) according to the manufacturer’s instructions. RT-PCR experiments were then carried out to visualize spliced and non-spliced product. Band intensity was quantified using ImageJ (NIH) and represents the mean ± SEM from three independent experiments.

### Dynamics of RNA splicing

We measured the kinetics of transcription and splicing recovery following release from drug-induced transcriptional arrest using a previously published protocol [37]. Briefly, control siRNA-treated cells and PRPF8-depleted cells were treated with DRB, a reversible inhibitor of transcriptional elongation [44]. Total RNA was then harvested at defined time-points following DRB removal and the time between synthesis of a new exon and the appearance of the splicing product of that exon was measured [37]. Cal51 cells were seeded at 1.5 × 10^5^cells/ml for control siRNA treatment and 2.5 × 10^5^ cells/ml for PRPF8 siRNA treatment in 2 ml DMEM in order to ensure an even number of cells for both conditions at the time of DRB treatment. The indicated siRNAs were reverse transfected with DharmaFECT according to the protocol above. Fifty-one hours post-transfection, 100 μM of DRB was added to cells and incubated for 3 hours to inhibit transcriptional elongation. Fifty-four hours post-transfection, media was removed, cells were washed twice with PBS, and 2 ml of new media was added. Cells were harvested at 5-minute intervals starting 0 minutes after DRB removal by direct lysis with RLT buffer (Qiagen) and subsequent vigorous scraping. Lysates were homogenized with QIAshredder columns (Qiagen) and RNA preparation with RNeasy kit (Qiagen) followed according to the manufacturer’s protocol. All subsequent qPCR results were normalized to non-DRB-treated cells for each condition (control siRNA-treated and PRPF8-depleted cells), which were harvested alongside the 0 minute time-point cells. Primer pairs used are indicated in Fig. 8 and were chosen to measure the dynamics of RNA splicing of introns that are either retained or exons that are differentially used as determined by RNA sequencing experiments. Primers spanning exon 4: intron 4 of *CDC23*, exon 10: intron 10 of *CDC20*, exon 19: intron 19 and exon 2: intron 2 of *ASPM*, and exon 19: intron 19 of *Separase* were used to measure recovery of transcription following DRB release. Primers spanning exon 3: intron 4 of *CDC23*, exon 9: intron 10 of *CDC20*, exon 18: intron 19 and exon 1: intron 2 of *ASPM*, and exon 18: intron 19 of *Separase* were used to measure the appearance of the partially spliced product following DRB release. Values represent mean ± SEM from one representative experiment.

### Analysis of the spliceosome iCLIP data

Spliceosome iCLIP datasets for three control-siRNA-treated and three *PRPF8*-depleted samples were generated using the previously described iCLIP protocol [61] with minor modifications. Briefly, spliceosome iCLIP was performed with the 12F5 antibody directed against SmB/B’ (Santa Cruz) at a concentration of 5 μg/ml and using mild stringency conditions (lysis buffer, 50 mM Tris–HCl ph 7.4, 0.1 M NaCl, 0.5 % Igepal CA-630, 0.05 % SDS, 0.25 % Na-deoxycholate; wash buffer, 50 mM Tris–HCl ph 7.4, 0.5 M NaCl, 0.5 % Igepal CA-630, 0.05 % SDS, 0.25 % Na-deoxycholate, 1 mM EDTA). These reduced stringency conditions allow co-purification of other snRNP-associated RBPs, leading to distinct peaks in the summarized genome-wide maps around splice junctions that are associated with individual RBP occupancies (M. Briese, C. Sibley, J. Ule, unpublished observation). A complete analysis of the spliceosome iCLIP method that characterizes individual peaks is beyond the scope of this manuscript, and will be reported elsewhere (J. Ule, personal communication).

Each biological replicate was sequenced twice as technical replicates, resulting in a total of 12 samples. Spliceosome iCLIP libraries contained a four-nucleotide indexing barcode, and five-nucleotide random barcode to allow removal of PCR duplicates. The obtained reads were mapped to the human genome (hg19) with Bowtie [62] and analyzed as described previously [27]. Bed files that contain information on spliceosome occupancy along the genome were generated as a result, and further used for downstream analysis. iCLIP counts were normalized by library size to account for variations in sequencing depth across samples. Similarly, for each junction, counts were divided by the maximum value across all libraries to allow for the comparison across different features.

### Analysis of the RNA-seq data

#### Datasets and mapping

The transcriptome of control siRNA-treated and PRPF8-depleted Cal51 cells was sequenced on an Illumina HiSeq2000 platform using 100 bp paired-end reads with RNA isolated from three and four independent depletion experiments, respectively. Due to the high quality of the reads, raw data were directly mapped to the human genome (Ensembl 66 [63]) using TopHat v.1.3.3 [64] with the options --max-multihits 1 --no-novel-juncs --min-isoform-fraction 0.0, and using the genome annotation as a guide.

#### Intron counts and normalized intron expression

Intron coordinates were obtained with custom scripts based on bedtools v.2.17.0 [65] by considering exons from transcripts annotated as protein coding in Ensembl 66. Overlapping exons were merged, and the longest exon or combination of exons was kept. Intronic expression levels were then obtained with dexseq-count (DEXSeq v.1.7.0 [66]) and normalized as follows:

$$ {\widehat{\mu}}_i=\frac{c_i/{l}_i}{c_g/{l}_g}\cdot \frac{10^9}{N} $$

where ${\widehat{\mu}}_i$ = normalized expressions for intron *i*, *c*_*i*_*=* counts for intron *i*, *l*_*i*_*=* length for intron *i*, *c*_*g*_*=* counts for gene *g*, *l*_*g*_*=* length for gene *g*, and *N =* library size.

#### Identification of differentially used exons

DEXSeq v.1.7.0 was used to identify genes with differential exon usage across the two studied conditions. Specific options include --aggregate=no for the preparation of the annotation and --paired=yes --stranded=no to count reads that overlap exons. An FDR threshold of 0.01 was used to assess the significance of the detected fold changes (Benjamini and Hochberg *p* value correction [67]).

#### Identification of retained introns

Differential intron usage was assessed with DEXSeq v.1.7.0 with the same options as indicated above. Due to the overall low number of intronic reads compared with exonic ones, library size factors were not inferred from intron counts, but from exon counts instead. Differentially used introns with a positive *log*_2_ fold change (FDR < 0.01, Benjamini and Hochberg *p* value correction) were defined as retained introns (RI), whilst those that did not fulfill this criterion were classified as non-retained (NRI). The most extreme intron retention events (i.e., the top 200) were selected for subsequent analyses based on fold change information.

Splice site strength was calculated with the MaxEntScan algorithm [33] and motif enrichment analysis was performed with Homer v.2 [68] using a common set of background sequences. The calculation of differential GC content in introns versus adjacent exons was performed similarly to [34] by discarding 20 nucleotides from each end of the introns and 3 nucleotides from the exons in order to account for splicing signals.

#### Gene level analyses

A gene was defined as expressed if it is detected above 1 FPKM in any given sample. Gene Ontology enrichment analyses were performed with DAVID [69] and WebGestalt [70] using an adjusted *p* value threshold of 0.01 (Benjamini and Yekutieli correction [71]) and the set of expressed genes as a background (*n* = 13,216). Differential gene expression was assessed with DESeq v.1.10.1 [72] and significant changes were evaluated following the criteria previously used for the analysis of differential splicing (FDR < 0.01, Benjamini and Hochberg correction).

#### Co-transcriptional splicing ratio

Co-transcriptional splicing was evaluated by comparing intronic expression levels for the first versus last introns of each transcript. Given the potential complexity introduced by alternative splicing events, this analysis was limited to genes with only one transcript annotated in Ensembl 66. Genes with less than two introns, those shorter than 300 bp (which would be missed in the size selection step during library preparation) and those that overlap with other genes in either strand were also discarded. Altogether, such filtering led to a final set of 2366 genes (i.e., transcripts) that could be considered in the analysis. Following the identification of the first and last introns of each transcript in the study set, a co-transcriptional splicing ratio was calculated using the formula detailed in Fig. 8.

### Data availability

RNA-seq data can be accessed from the ArrayExpress database with the following accession number, E-MTAB-3021.

## Additional files

Additional file 1:
**Validation of PRPF8 depletion.** **a** Validation of PRPF8 depletion by qRTPCR. Results are plotted relative to RNA levels in Control siRNA-treated cells, assigned an arbitrary value of 1, and show the mean of triplicate repeats from ten independent experiments ± standard error of the mean. **b** Exposure to the specific but not control siRNAs efficiently depletes PRPF8 protein in Cal51 cells as detected by western blotting with the indicated antibodies. **c**, **d** Characterization of spliceosome iCLIP experiments. UV-crosslinked Cal51 cells were lysed and subjected to partial RNase I digestion (low, final dilution of 1:100,000; high, dilution of 1:5000). Spliceosomal RNPs were immunopurified with anti-SmB/B’ antibody, RNA was 5′ end radiolabeled, and RNPs subjected to denaturing gel electrophoresis and nitrocellulose transfer, an autoradiogram of which is shown. Each lane represents an independent depletion experiment. The interrupted line indicates the area on the nitrocellulose membrane cut out for purification of crosslinked RNP complexes. **e** Library stats giving the ratio of unique cDNA counts to total cDNA counts for each of the libraries sequenced. The average from three experimental replicates and two technical replicates for each condition is indicated. (PDF 190 kb) Additional file 2:
**Genome-wide decreases in spliceosome assembly at intron–exon junctions following PRPF8 depletion are independent of expression levels and intron retention.** **a**, **b** Genome-wide decreases in spliceosome assembly following PRPF8 depletion are independent of expression levels. Spliceosome iCLIP plots around 5′ (**a**) and 3′ (**b**) splice site junctions were generated after excluding differentially expressed genes between the PRPF8-depleted and control siRNA-treated populations of cells from the analysis. **c**, **d** Spliceosome iCLIP plots around 5′ (**c**) and 3′ (**d**) splice site junctions were generated using the subset of retained introns (*top panels*) and the subset of non-retained introns (*bottom panels*) in control siRNA-treated and PRPF8-depleted cells. Relevant peaks around 5′ and 3′ splice site junctions that change in response to PRPF8 depletion are indicated by *red arrowheads*. (PDF 128 kb) Additional file 3:
**Altered splicing of transcripts encoding proteins required for mitotic progression after PRPF8 depletion.** **a** RT-PCR analysis of RNA from PRPF8-depleted cells reveals a variety of splicing defects in transcripts that encode critical factors required for mitotic progression, including a retained intron (*NUDC*) and skipped exons (*ASPM* and *SKA2*). RTPCR analysis of *ASPM*, *NUDC*, and *SKA2* using the primers indicated in the schematic are shown alongside the corresponding sashimi plots (control siRNA in *black*, PRPF8 siRNA in *red*). The splicing defects are indicated by *arrowheads* and counts spanning splice site junctions that change in the skipped exons are also indicated. **b**, **c** Splicing alterations in mitotic genes do not normally occur during transit through mitosis. Cal51 cells treated with nocodazole for 4 or 15 hours to induce mitotic arrest were monitored for mitotic index (**b**), and analyzed by qRT-PCR for splicing alterations previously observed following PRPF8 depletion (see Fig. 2) (**c**). Plots are relative to RNA levels in control siRNA-treated cells, assigned an arbitrary value of 1, and show the mean of triplicate readings from three independent experiments ± standard error of the mean (SEM). Mitotic index plots also show the mean from three independent experiments ± SEM. (PDF 264 kb) Additional file 4:
**Validation of mitotic arrest following PRPF8 depletion with two independent siRNAs.** **a** U2OS osteosarcoma cells depleted of PRPF8 using two independent siRNAs and monitored by phase-contrast time-lapse microscopy spend significantly longer in mitosis (measured from nuclear envelope breakdown (NEB) to chromatin decondensation), as opposed to control cells, in which mitosis lasts, on average, <60 min. Statistically significant pairwise comparisons are shown (****p* < 0.001). **b** Introduction of properly spliced and processed mRNA into Control siRNA-treated cells has no effect on mitotic index. Control siRNA-treated cells were transfected with properly spliced and processed mRNA purified from whole cells and analyzed for mitotic index. Plots represent the mean from two independent experiments ± standard error of the mean. *n.s*. not significant. (PDF 77 kb) Additional file 5:
**Depletion of Complex B components that interact directly with PRPF8 recapitulate defects in mitotic progression.** **a** Independent depletion of several different components of the three major spliceosome subcomplexes (A, B, and C) used in Fig. 4 was verified by qRT-PCR. Plots are relative to RNA levels in control siRNA-treated cells, assigned an arbitrary value of 1, and show the mean of triplicate readings from at least three independent experiments ± standard error of the mean. **b** Bar graph depicting time spent in mitosis, measured from nuclear envelope breakdown to anaphase, obtained from live-cell imaging of U2OS cells stably expressing GFP-tagged histone H2B and depleted of spliceosome subcomplexes (A, B and C). For simplicity, cells are classified as spending either less than 60 min in mitosis (*black bars*), or >60 minutes (*red bars*). **c** Percentage of mitotic cells depleted of spliceosome subcomplexes (A, B, and C) with defects in chromosome alignment and/or segregation, as shown in Fig. 4. (PDF 155 kb) Additional file 6:
**Inefficiently spliced introns and exons that are differentially used do not have weaker 3′ splice sites.** **a** Inefficiently spliced introns do not have weaker 3′ splice sites. A set of 200 retained introns (*RI*) were selected based on fold change differences (see “Materials and methods”), and non-retained introns (*NRI*) within the same set of genes were used as a contrast. A 3′ splice site strength analysis was then carried out as described in “Materials and methods” for each subset. **b** Motif enrichment analysis on the same set of genes shows that the most frequently identified motifs correspond to the consensus 3′ splice site sequences for both retained (RI) and non-retained introns (NRI) and there is no significant change in the percentage of targets with such motifs. **c** Differentially used exons do not have weaker 3′ splice sites. A 3′ splice site strength analysis was carried out as described in “Materials and methods” for each subset. (PDF 63 kb) Additional file 7:
**Enhancement of 3′ splice-site strength renders the** ***CDC20*** **mini-gene resistant to PRPF8 depletion.** **a** A mini-gene containing a full-length intron flanked by two exons within the *CDC20* gene. This particular intron is retained following PRPF8 depletion as determined by RNA-sequencing experiments and the corresponding coverage plot is shown. The 3′ splice site encompassing the intron–exon boundary is weaker than the corresponding consensus sequence and the sequence of 3′ splice mutant constructs are indicated with changes marked in *red*. **b**, **c** Enhancement of 3′ splice site strength renders the *CDC20* mini-gene resistant to PRPF8 depletion. PRPF8 depletion strongly suppresses removal of this intron in the *CDC20* mini-gene (*left panel*). Strengthening of the polypyrimidine tract allows efficient removal of this intron from the *CDC20* mini-gene in PRPF8-deficient cells (*middle panel*). Plots in (**c**) represent quantification of band intensity of spliced and unspliced product using ImageJ (NIH) and represent the mean percentage of spliced mRNA ± standard error of the mean from three independent experiments. Statistically significant pairwise comparisons are indicated (**p* < 0.05, ***p* < 0.01). *n.s*. not significant, *WT* wild type. (PDF 135 kb) Additional file 8:
**PRPF8 depletion alters the dynamics of RNA splicing during transcription.** **a** Raw data used to calculate co-transcriptional splicing ratio in Fig. 8a is shown. Normalized intron expression for first introns (f_k_, *orange*) and last introns (f_I_, *blue*) is shown for each independent depletion experiment (three for control siRNA, and four for PRPF8 siRNA). **b** The 5′ splice site strength of first and last introns are similar. The 5′ splice site strength was analyzed as described in “Materials and methods” for the first and last introns in the 2380 genes considered for the analysis of the co-transcriptional splicing ratio. The *p* value is indicated. **c** PRPF8 depletion alters the dynamics of RNA splicing during transcription. The kinetics of transcription and splicing recovery of the *Separase* gene following release from drug-induced transcriptional arrest were measured in control siRNA-treated and PRPF8-depleted cells in a similar fashion to that shown in Fig. 8d. (PDF 109 kb) Additional file 9:
**Transcription initiation is similar in control siRNA-treated and PRPF8-depleted cells.** **a** Delay between detection of the unspliced nascent transcript and the partially spliced transcript for *UBE2R2* is similar in both PRPF8-depleted and control siRNA-treated cells. Kinetics of transcription and splicing recovery for *UBE2R2*, a gene whose splicing was unaffected by PRPF8 depletion as determined in our RNA-sequencing data, following release from drug-induced transcriptional arrest were measured in control siRNA-treated and PRPF8-depleted cells in a similar fashion to that shown in Fig. 8d. **b** Transcription of the first exon–intron junction of the four target genes following release from DRB in PRPF8-depleted and control siRNA-treated cells was measured. Primer pairs used span exon 1: intron 1 and thus measure transcription initiation following release from drug-induced transcriptional arrest. (PDF 198 kb)

## Abbreviations

bp: base pair
DMEM: Dulbecco’s modified Eagle medium
DRB: 5,6-dichlorobenzimidazole 1-β-D-ribofuranoside
FDR: false discovery rate
iCLIP: UV crosslinking and immunoprecipitation
NRI: non-retained intron
PBS: phosphate-buffered saline
PBST: PBS with 0.1 % Triton X-100
pre-mRNA: precursor messenger RNA
qRT-PCR: quantitative RT-PCR
RBP: RNA-binding protein
RI: retained intron
RNAi: RNA interference
RNP: ribonucleoprotein
RT-PCR: Reverse transcription-polymerase chain reaction
SEM: standard error of the mean
siRNA: short interfering RNA
snRNP: small nuclear ribonucleoprotein complex
U snRNA: uridine-rich small nuclear RNA
