# Supplementary material for: Regulation of constitutive and alternative mRNA splicing across the human transcriptome by PRPF8 is determined by 5′ splice site strength
Source: Genome Biol. 2015 Sep 21;16(1):201. doi: 10.1186/s13059-015-0749-3 (PMC4578845; doi:10.1186/s13059-015-0749-3)
Supplement: Additional file 1: — Validation of PRPF8 depletion. a Validation of PRPF8 depletion by qRTPCR. Results are plotted relative to RNA levels in Control siRNA-treated cells, assigned an arbitrary value of 1, and show the mean of triplicate repeats from ten independent experiments ± standard error of the mean. b Exposure to the specific but not control siRNAs efficiently depletes PRPF8 protein in Cal51 cells as detected by western blotting with the indicated antibodies. c, d Characterization of spliceosome iCLIP experiments. UV-crosslinked Cal51 cells were lysed and subjected to partial RNase I digestion (low, final dilution of 1:100,000; high, dilution of 1:5000). Spliceosomal RNPs were immunopurified with anti-SmB/B’ antibody, RNA was 5′ end radiolabeled, and RNPs subjected to denaturing gel electrophoresis and nitrocellulose transfer, an autoradiogram of which is shown. Each lane represents an independent depletion experiment. The interrupted line indicates the area on the nitrocellulose membrane cut out for purification of crosslinked RNP complexes. e Library stats giving the ratio of unique cDNA counts to total cDNA counts for each of the libraries sequenced. The average from three experimental replicates and two technical replicates for each condition is indicated. (PDF 190 kb) [file 13059_2015_749_MOESM1_ESM.pdf]

Additional file 1

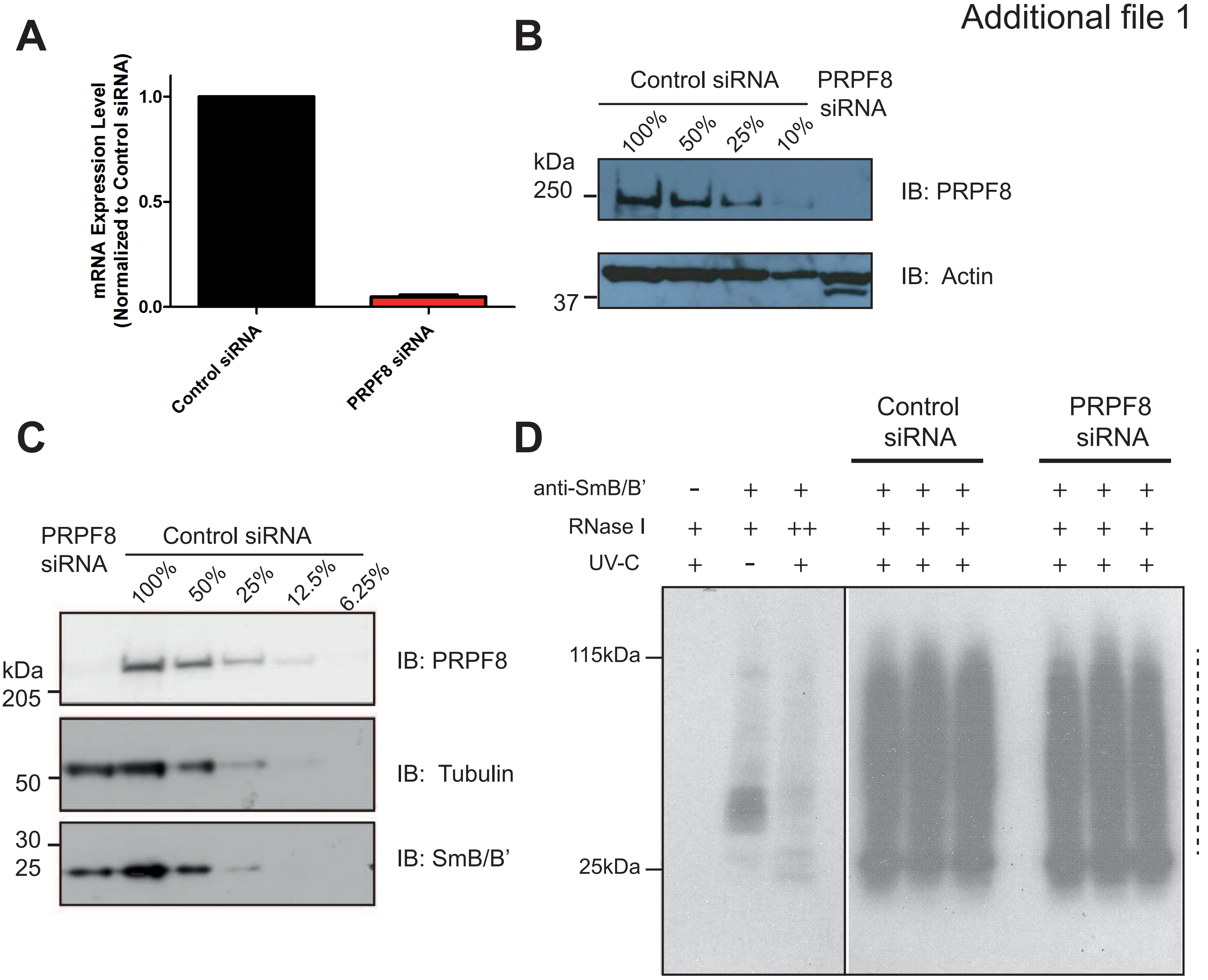

E

	Unique counts	Total counts	Ratio
siNS_1	844433	3833910	4.54
siNS_2	1224640	5575620	4.55
siNS_3	2575470	12062600	4.68
siNS_4	10057600	49189100	4.89
siNS_5	4025010	19260900	4.79
siNS_6	6002830	28812800	4.80
Average	4121664	19789155	
siPRPF8_1	2955440	11755800	3.98
siPRPF8_2	3778840	14707300	3.89
siPRPF8_3	5574360	22540000	4.04
siPRPF8_4	13480500	56538300	4.19
siPRPF8_5	2395680	8915570	3.72
siPRPF8_6	5042260	20590100	4.08
Average	5537847	22507845	
