# Supplementary material for: Regulation of constitutive and alternative mRNA splicing across the human transcriptome by PRPF8 is determined by 5′ splice site strength
Source: Genome Biol. 2015 Sep 21;16(1):201. doi: 10.1186/s13059-015-0749-3 (PMC4578845; doi:10.1186/s13059-015-0749-3)

Additional file 2

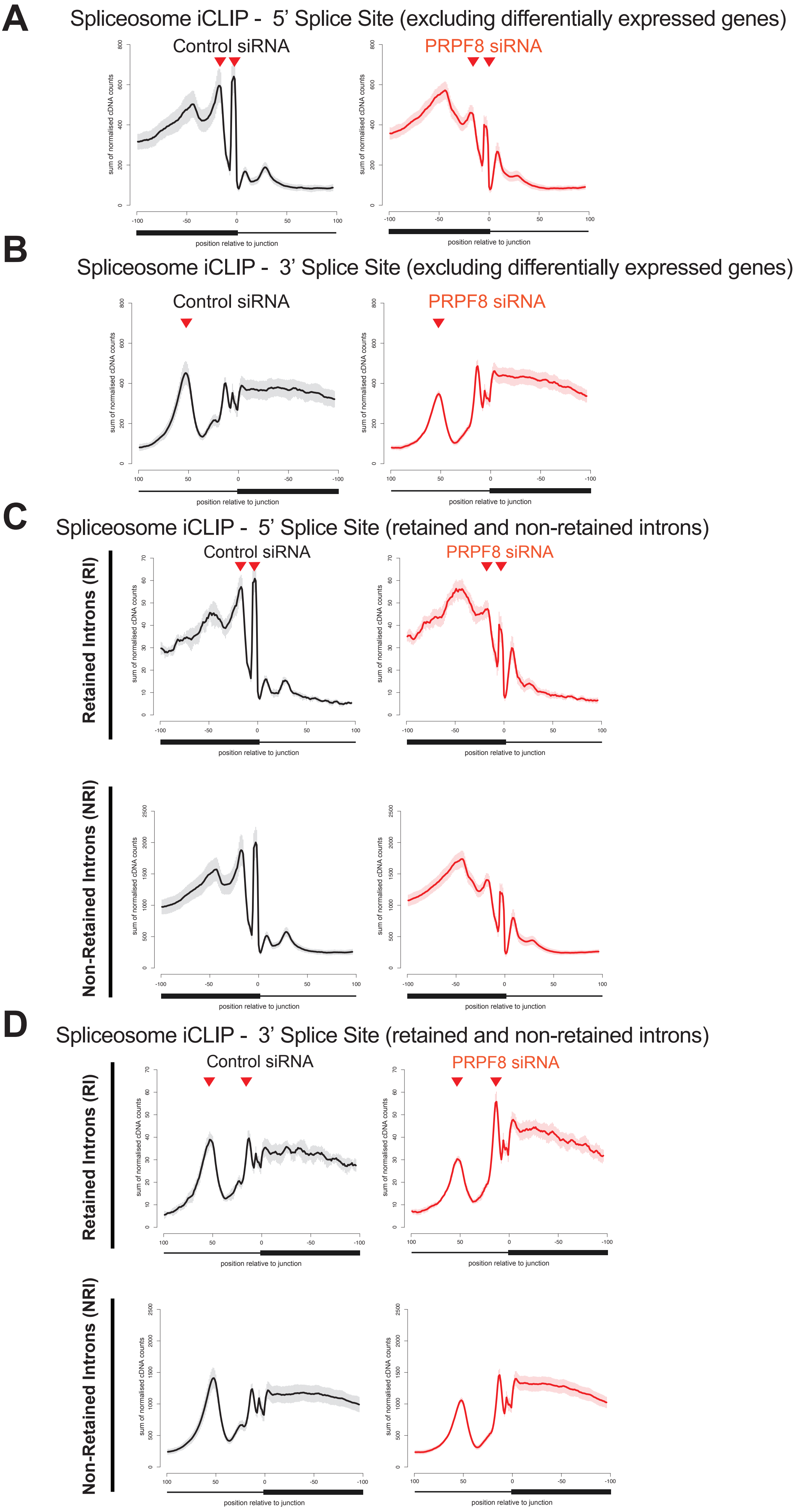

Supplement: Additional file 2: — Genome-wide decreases in spliceosome assembly at intron–exon junctions following PRPF8 depletion are independent of expression levels and intron retention. a, b Genome-wide decreases in spliceosome assembly following PRPF8 depletion are independent of expression levels. Spliceosome iCLIP plots around 5′ (a) and 3′ (b) splice site junctions were generated after excluding differentially expressed genes between the PRPF8-depleted and control siRNA-treated populations of cells from the analysis. c, d Spliceosome iCLIP plots around 5′ (c) and 3′ (d) splice site junctions were generated using the subset of retained introns (top panels) and the subset of non-retained introns (bottom panels) in control siRNA-treated and PRPF8-depleted cells. Relevant peaks around 5′ and 3′ splice site junctions that change in response to PRPF8 depletion are indicated by red arrowheads. (PDF 128 kb) [file 13059_2015_749_MOESM2_ESM.pdf]
