# Supplementary figures and images for: Regulation of constitutive and alternative mRNA splicing across the human transcriptome by PRPF8 is determined by 5′ splice site strength
Source: Genome Biol. 2015 Sep 21;16(1):201. doi: 10.1186/s13059-015-0749-3 (PMC4578845; doi:10.1186/s13059-015-0749-3)

**A**

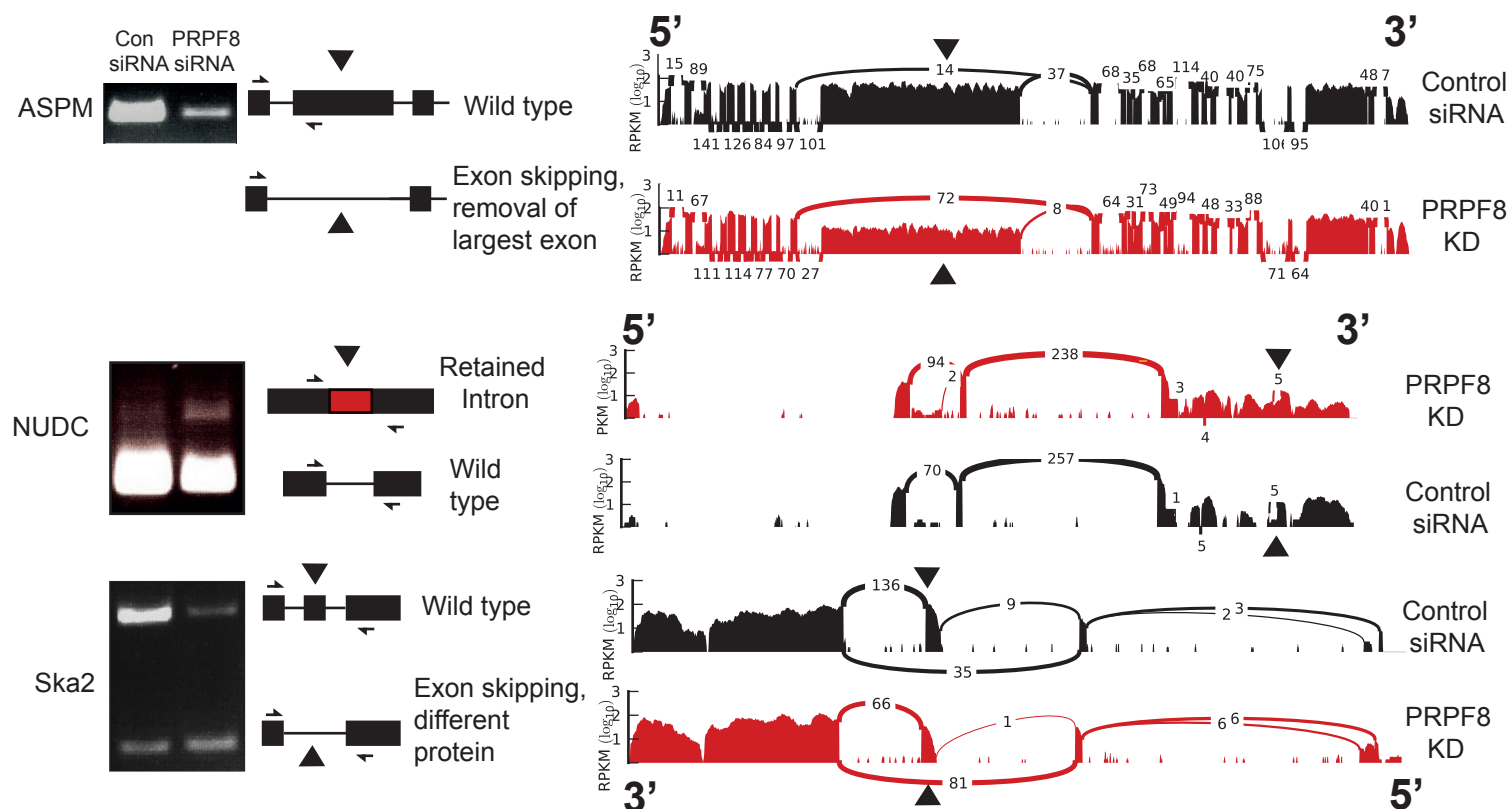

**B**

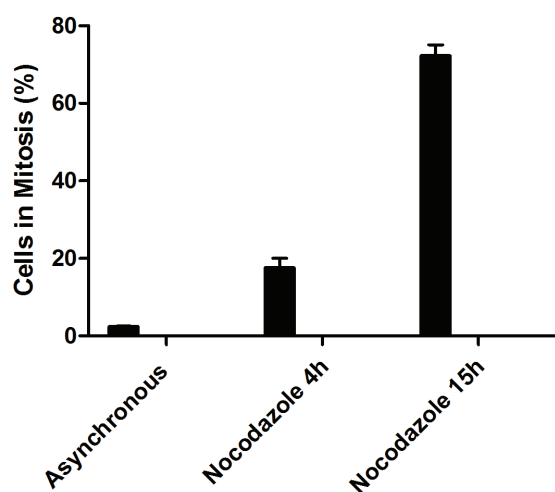

**C**

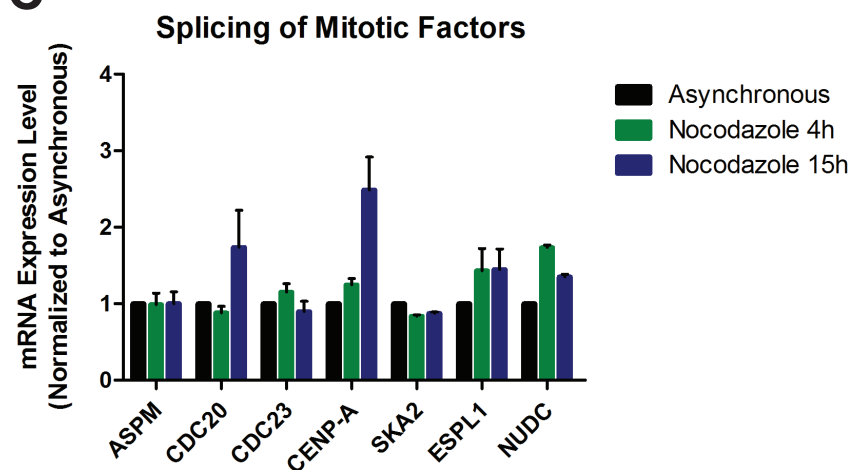

Supplement: Additional file 3: — Altered splicing of transcripts encoding proteins required for mitotic progression after PRPF8 depletion. a RT-PCR analysis of RNA from PRPF8-depleted cells reveals a variety of splicing defects in transcripts that encode critical factors required for mitotic progression, including a retained intron (NUDC) and skipped exons (ASPM and SKA2). RTPCR analysis of ASPM, NUDC, and SKA2 using the primers indicated in the schematic are shown alongside the corresponding sashimi plots (control siRNA in black, PRPF8 siRNA in red). The splicing defects are indicated by arrowheads and counts spanning splice site junctions that change in the skipped exons are also indicated. b, c Splicing alterations in mitotic genes do not normally occur during transit through mitosis. Cal51 cells treated with nocodazole for 4 or 15 hours to induce mitotic arrest were monitored for mitotic index (b), and analyzed by qRT-PCR for splicing alterations previously observed following PRPF8 depletion (see Fig. 2) (c). Plots are relative to RNA levels in control siRNA-treated cells, assigned an arbitrary value of 1, and show the mean of triplicate readings from three independent experiments ± standard error of the mean (SEM). Mitotic index plots also show the mean from three independent experiments ± SEM. (PDF 264 kb) [file 13059_2015_749_MOESM3_ESM.pdf]

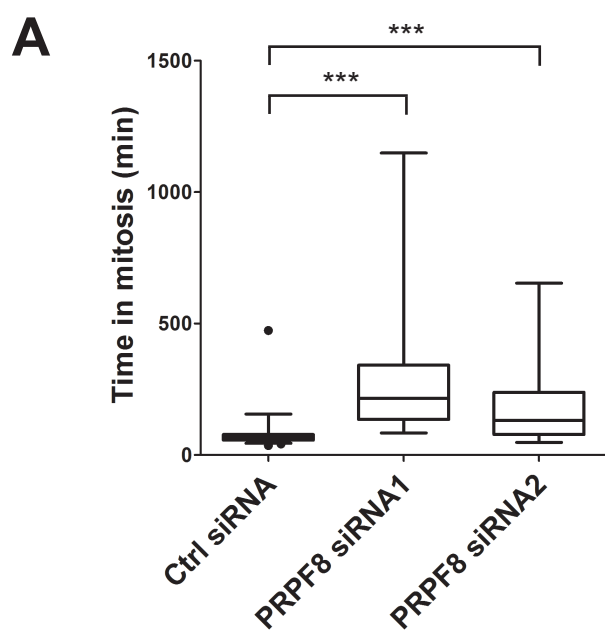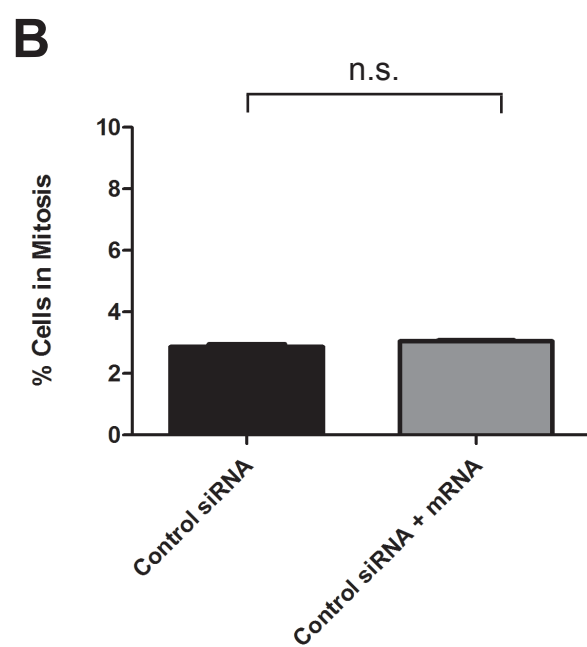

Supplement: Additional file 4: — Validation of mitotic arrest following PRPF8 depletion with two independent siRNAs. a U2OS osteosarcoma cells depleted of PRPF8 using two independent siRNAs and monitored by phase-contrast time-lapse microscopy spend significantly longer in mitosis (measured from nuclear envelope breakdown (NEB) to chromatin decondensation), as opposed to control cells, in which mitosis lasts, on average, <60 min. Statistically significant pairwise comparisons are shown (***p < 0.001). b Introduction of properly spliced and processed mRNA into Control siRNA-treated cells has no effect on mitotic index. Control siRNA-treated cells were transfected with properly spliced and processed mRNA purified from whole cells and analyzed for mitotic index. Plots represent the mean from two independent experiments ± standard error of the mean. n.s. not significant. (PDF 77 kb) [file 13059_2015_749_MOESM4_ESM.pdf]

**A****Depletion Efficiency for experiments in Figure 4**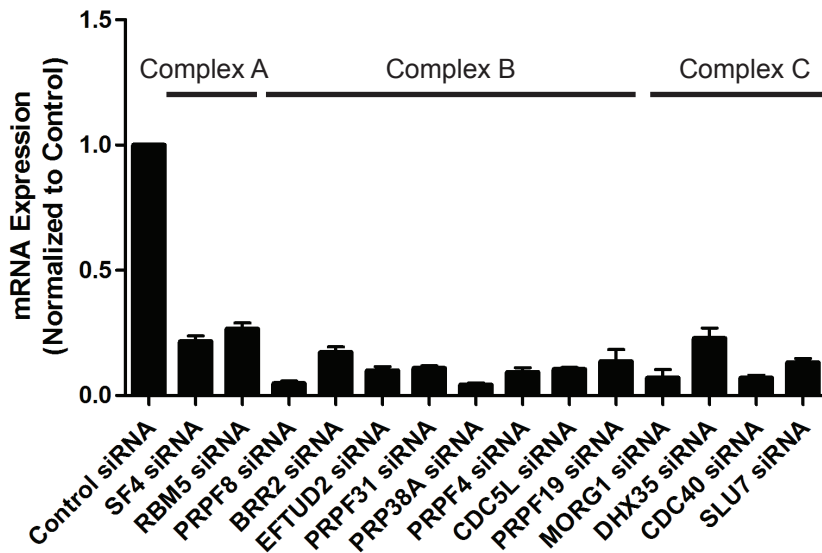**B****Time in Mitosis**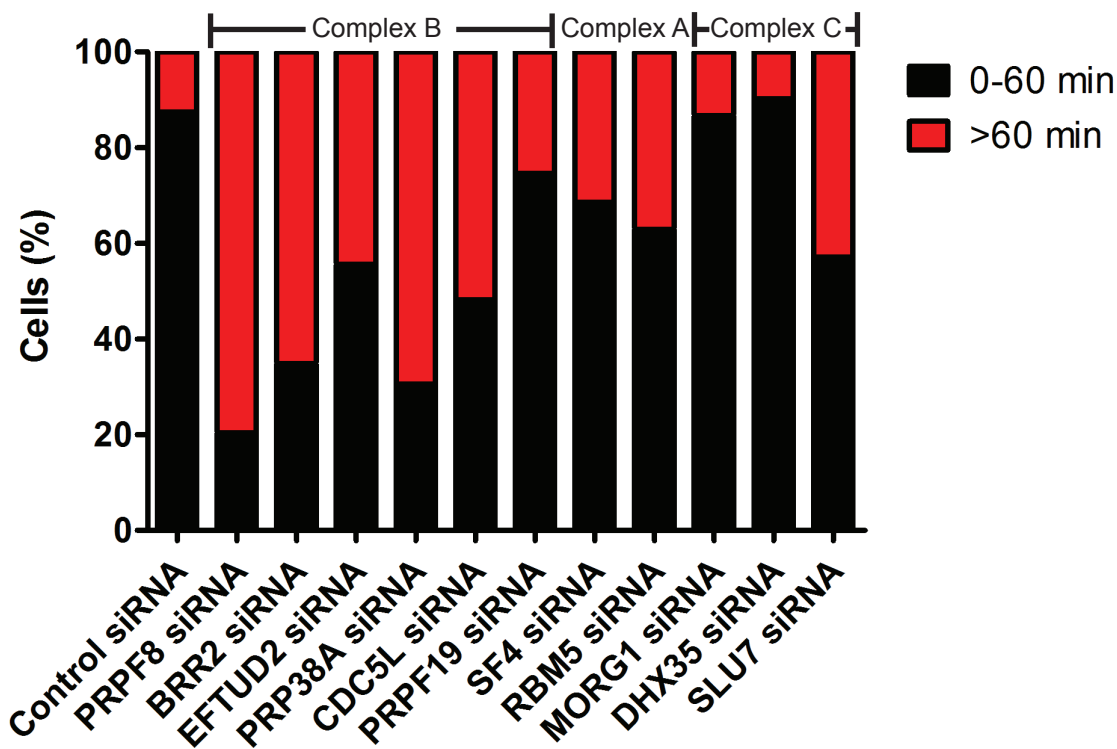**C****Abnormal Mitosis**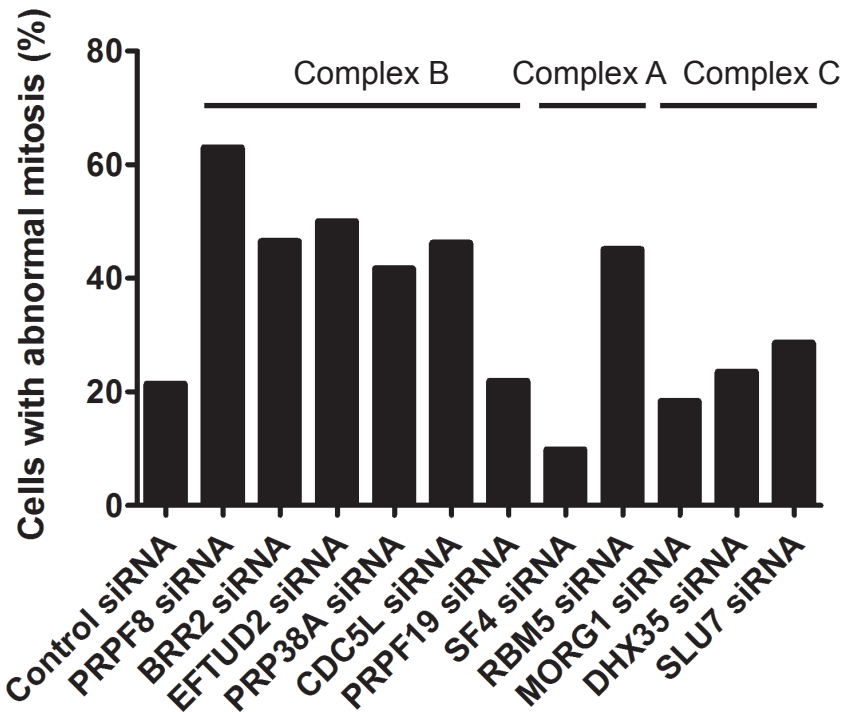

Supplement: Additional file 5: — Depletion of Complex B components that interact directly with PRPF8 recapitulate defects in mitotic progression. a Independent depletion of several different components of the three major spliceosome subcomplexes (A, B, and C) used in Fig. 4 was verified by qRT-PCR. Plots are relative to RNA levels in control siRNA-treated cells, assigned an arbitrary value of 1, and show the mean of triplicate readings from at least three independent experiments ± standard error of the mean. b Bar graph depicting time spent in mitosis, measured from nuclear envelope breakdown to anaphase, obtained from live-cell imaging of U2OS cells stably expressing GFP-tagged histone H2B and depleted of spliceosome subcomplexes (A, B and C). For simplicity, cells are classified as spending either less than 60 min in mitosis (black bars), or >60 minutes (red bars). c Percentage of mitotic cells depleted of spliceosome subcomplexes (A, B, and C) with defects in chromosome alignment and/or segregation, as shown in Fig. 4. (PDF 155 kb) [file 13059_2015_749_MOESM5_ESM.pdf]

**A**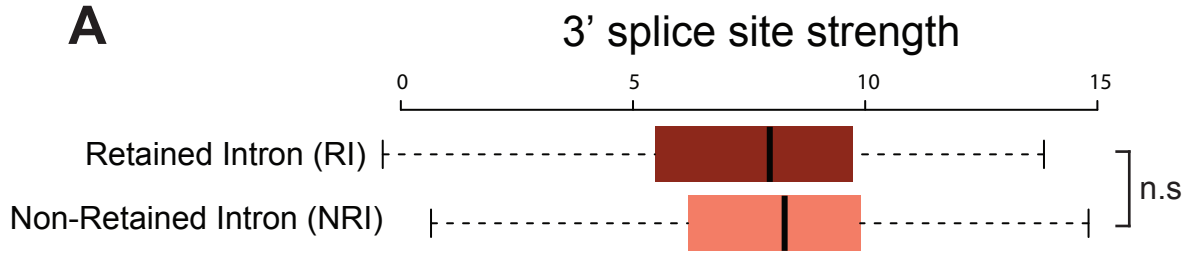**B**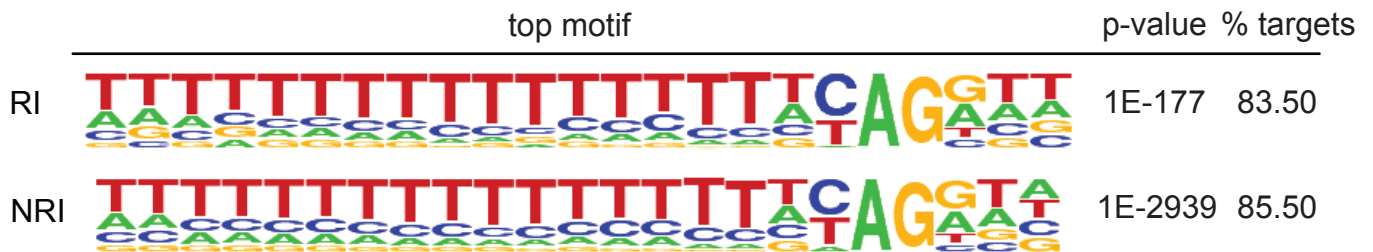**C**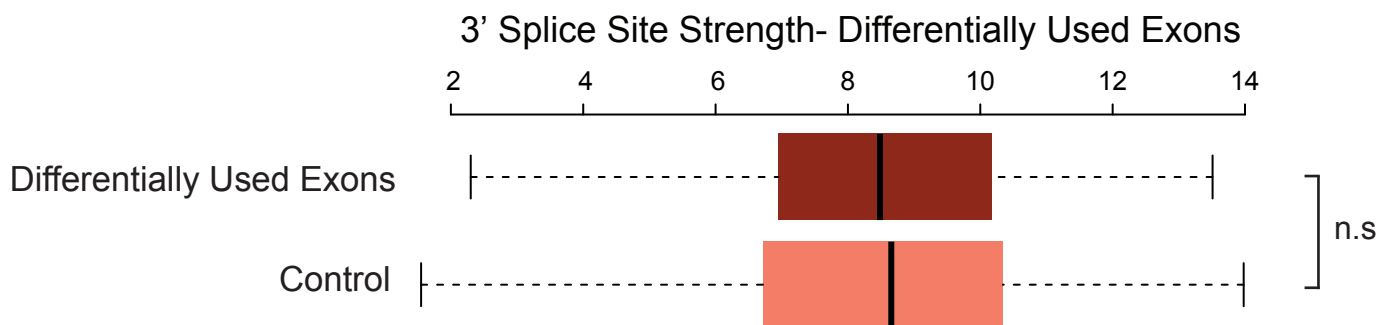

Supplement: Additional file 6: — Inefficiently spliced introns and exons that are differentially used do not have weaker 3′ splice sites. a Inefficiently spliced introns do not have weaker 3′ splice sites. A set of 200 retained introns (RI) were selected based on fold change differences (see “Materials and methods”), and non-retained introns (NRI) within the same set of genes were used as a contrast. A 3′ splice site strength analysis was then carried out as described in “Materials and methods” for each subset. b Motif enrichment analysis on the same set of genes shows that the most frequently identified motifs correspond to the consensus 3′ splice site sequences for both retained (RI) and non-retained introns (NRI) and there is no significant change in the percentage of targets with such motifs. c Differentially used exons do not have weaker 3′ splice sites. A 3′ splice site strength analysis was carried out as described in “Materials and methods” for each subset. (PDF 63 kb) [file 13059_2015_749_MOESM6_ESM.pdf]

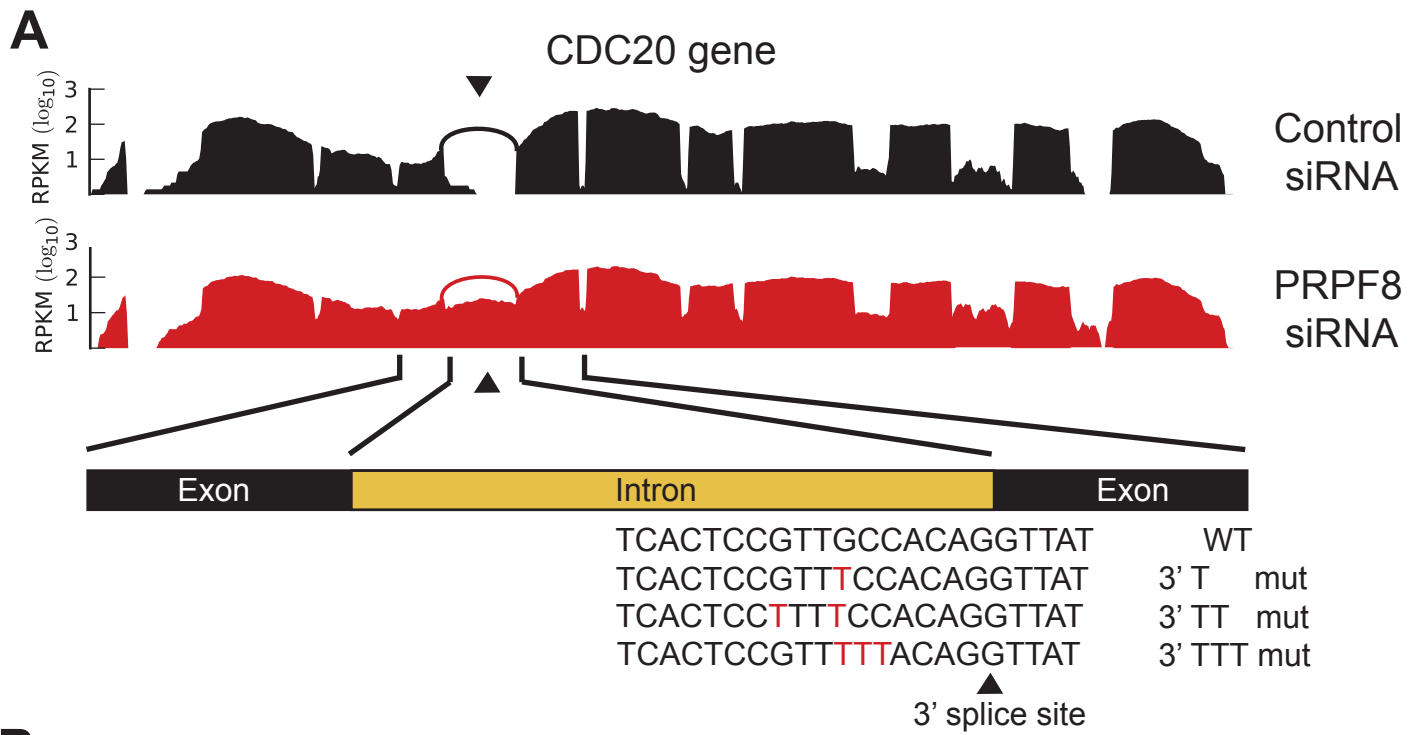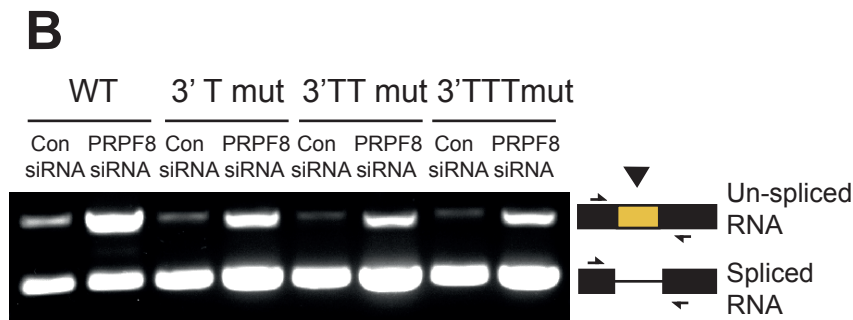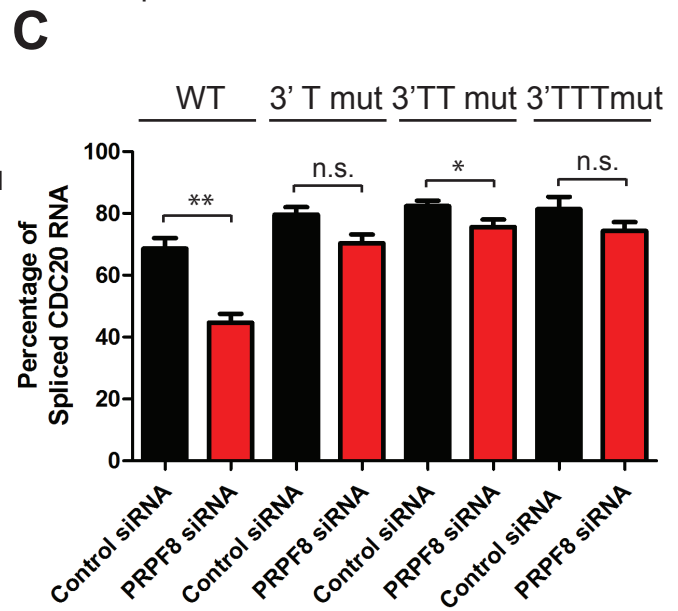

Supplement: Additional file 7: — Enhancement of 3′ splice-site strength renders the CDC20 mini-gene resistant to PRPF8 depletion. a A mini-gene containing a full-length intron flanked by two exons within the CDC20 gene. This particular intron is retained following PRPF8 depletion as determined by RNA-sequencing experiments and the corresponding coverage plot is shown. The 3′ splice site encompassing the intron–exon boundary is weaker than the corresponding consensus sequence and the sequence of 3′ splice mutant constructs are indicated with changes marked in red. b, c Enhancement of 3′ splice site strength renders the CDC20 mini-gene resistant to PRPF8 depletion. PRPF8 depletion strongly suppresses removal of this intron in the CDC20 mini-gene (left panel). Strengthening of the polypyrimidine tract allows efficient removal of this intron from the CDC20 mini-gene in PRPF8-deficient cells (middle panel). Plots in (c) represent quantification of band intensity of spliced and unspliced product using ImageJ (NIH) and represent the mean percentage of spliced mRNA ± standard error of the mean from three independent experiments. Statistically significant pairwise comparisons are indicated (*p < 0.05, **p < 0.01). n.s. not significant, WT wild type. (PDF 135 kb) [file 13059_2015_749_MOESM7_ESM.pdf]

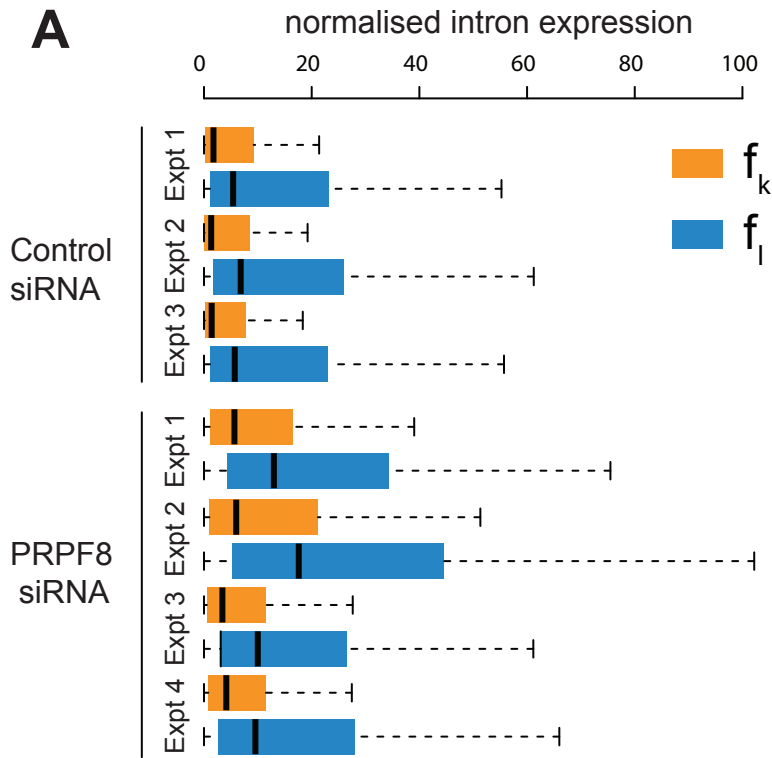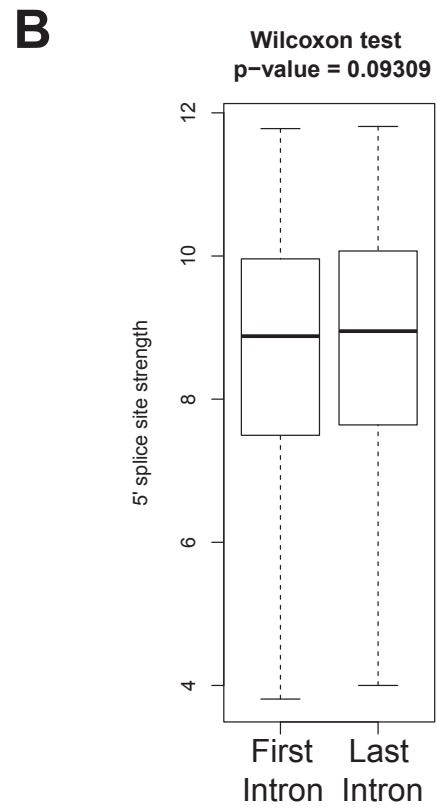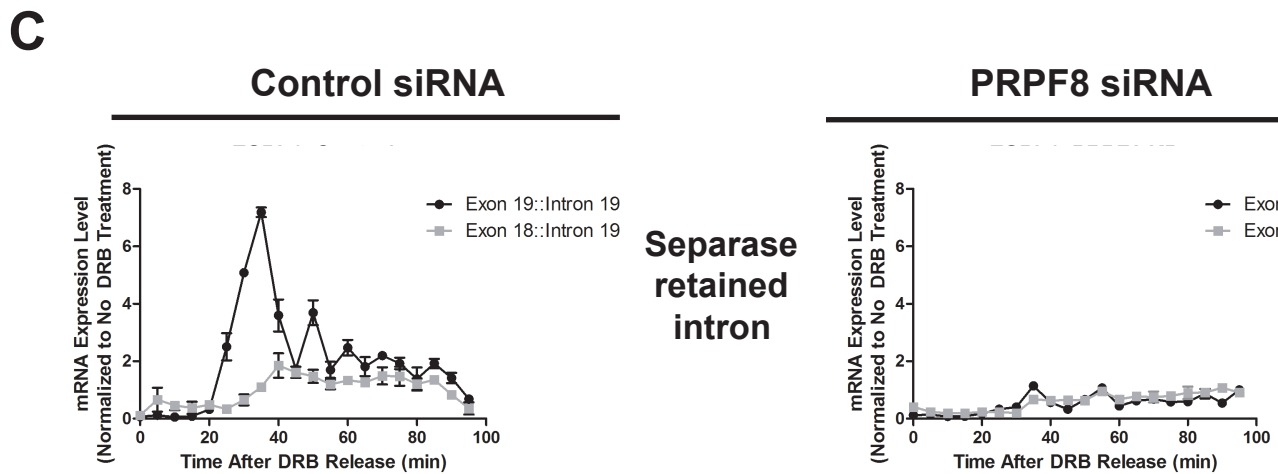

Supplement: Additional file 8: — PRPF8 depletion alters the dynamics of RNA splicing during transcription. a Raw data used to calculate co-transcriptional splicing ratio in Fig. 8a is shown. Normalized intron expression for first introns (fk, orange) and last introns (fI, blue) is shown for each independent depletion experiment (three for control siRNA, and four for PRPF8 siRNA). b The 5′ splice site strength of first and last introns are similar. The 5′ splice site strength was analyzed as described in “Materials and methods” for the first and last introns in the 2380 genes considered for the analysis of the co-transcriptional splicing ratio. The p value is indicated. c PRPF8 depletion alters the dynamics of RNA splicing during transcription. The kinetics of transcription and splicing recovery of the Separase gene following release from drug-induced transcriptional arrest were measured in control siRNA-treated and PRPF8-depleted cells in a similar fashion to that shown in Fig. 8d. (PDF 109 kb) [file 13059_2015_749_MOESM8_ESM.pdf]

**A****Control siRNA**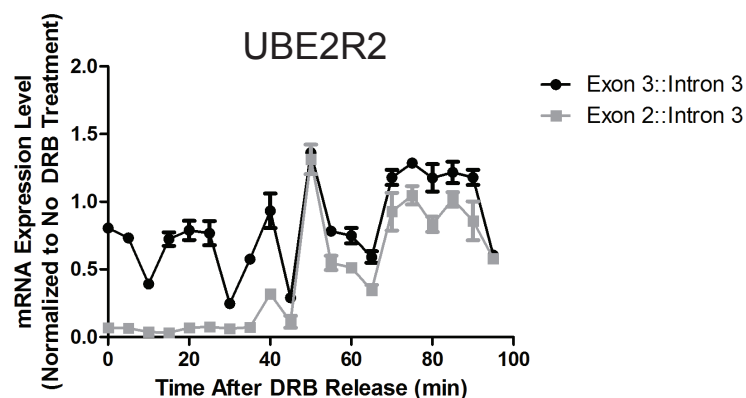**PRPF8 siRNA**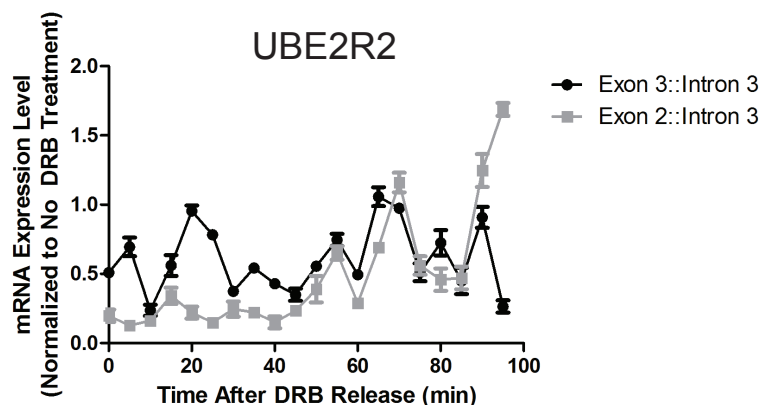**B****Transcription of first exon-intron junction**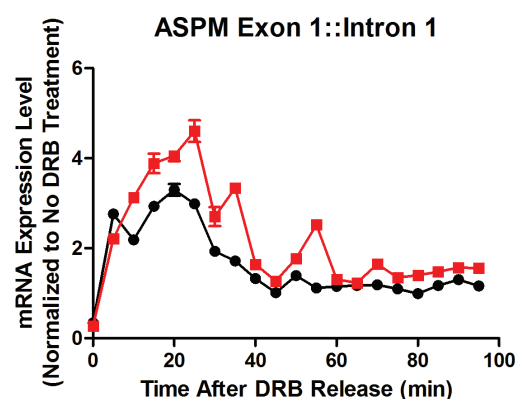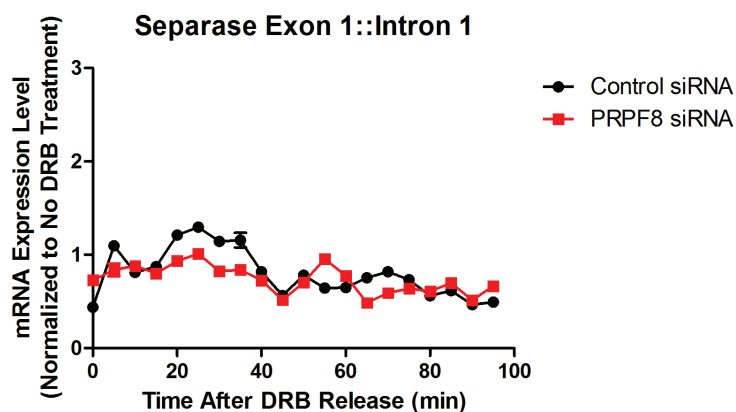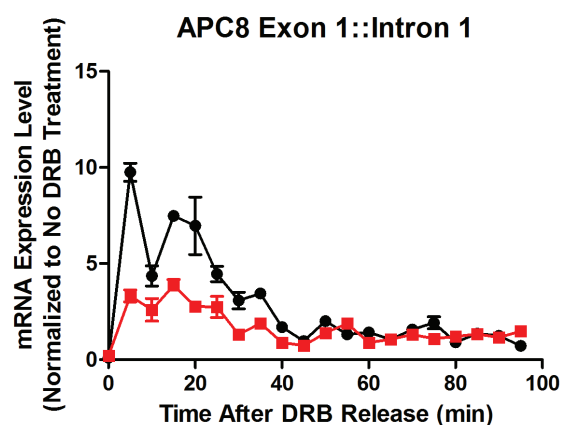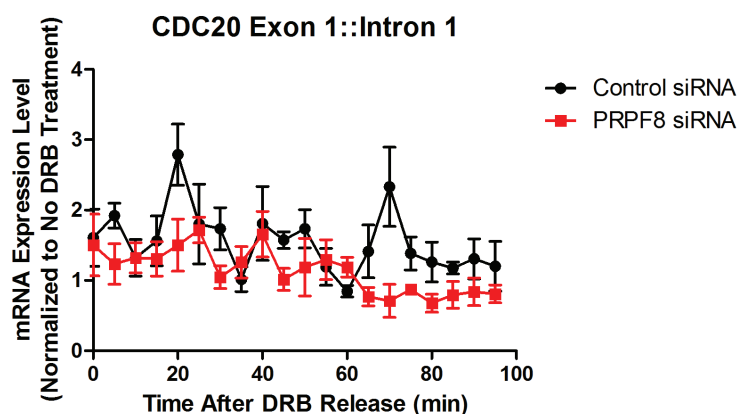

Supplement: Additional file 9: — Transcription initiation is similar in control siRNA-treated and PRPF8-depleted cells. a Delay between detection of the unspliced nascent transcript and the partially spliced transcript for UBE2R2 is similar in both PRPF8-depleted and control siRNA-treated cells. Kinetics of transcription and splicing recovery for UBE2R2, a gene whose splicing was unaffected by PRPF8 depletion as determined in our RNA-sequencing data, following release from drug-induced transcriptional arrest were measured in control siRNA-treated and PRPF8-depleted cells in a similar fashion to that shown in Fig. 8d. b Transcription of the first exon–intron junction of the four target genes following release from DRB in PRPF8-depleted and control siRNA-treated cells was measured. Primer pairs used span exon 1: intron 1 and thus measure transcription initiation following release from drug-induced transcriptional arrest. (PDF 198 kb) [file 13059_2015_749_MOESM9_ESM.pdf]
